# Dynamic functional connectivity variability may explain hypoxia-induced cognitive impairment at high-altitude

**DOI:** 10.1016/j.isci.2026.117015

**Published:** 2026-08-03

**Authors:** Siyao Zeng, Sijia Guo, Yang Zhou, Jiebing He, Yang Ou, Tingting Yang, Jia Wang, Wensheng Zhai, Baojuan Li, Jie Liu, Wenjing Luo, Xiaoming Chen

**Affiliations:** 1 The Ministry of Education Key Laboratory of Hazard Assessment and Control in Special Operational Environments, Shaanxi Provincial Key Laboratory of Environmental Health Hazard Assessment and Protection, Shaanxi Provincial Key Laboratory of Free Radical Biology and Medicine, Department of Occupational and Environmental Health, School of Public Health, Fourth Military Medical University, Xi’an, Shaanxi 710032, China; 2 Xijing Hospital, Fourth Military Medical University, Xi’an, Shaanxi 710032, China; 3 School of Biomedical Engineering, Fourth Military Medical University, Xi’an, Shaanxi 710032, China; 4 The General Hospital of PLA Xizang Military Area Command, Lhasa, Xizang 850007, China

**Keywords:** high altitude, cognitive impairments, dynamic functional connectivity, magnetic resonance imaging

## Abstract

Long-term exposure to a high-altitude (HA) hypoxic environment induces cognitive impairments, yet the underlying temporal mechanisms remain elusive. This longitudinal study investigated brain functional alterations associated with cognitive changes in 49 college freshmen relocated from sea level to Tibet, with comprehensive cognitive assessments and magnetic resonance imaging (MRI) at baseline and 2- and 4-year follow-ups. Resting-state fMRI quantified changes in regional homogeneity (ReHo), amplitude of low-frequency fluctuations (ALFF)/fractional ALFF (fALFF), static functional connectivity (sFC), and dynamic FC (dFC). Behavioral data confirmed persistent cognitive deficits, while neuroimaging analyses revealed biphasic patterns (initial suppression then partial/full recovery) in ReHo, ALFF/fALFF, and sFC. Notably, dFC variability in the right orbital middle frontal gyrus (ORBmid.R) and Heschl’s gyrus (HES.R) increased at 2 years and remained elevated, with this alteration strongly correlated with cognitive changes. Our findings highlight that elevated dFC variability in two brain regions is a key contributor to chronic hypoxia-induced cognitive impairments.

## Highlights

• ALFF/fALFF exhibited initial decreases followed by full recovery during HA exposure
• ReHo and sFC decreased initially and then partially recovered during HA exposure
• dFC variability increased initially and then partially recovered during HA exposure
• Increased dFC variability between ORBmid.R-HES.R correlated with cognitive changes

## Graphical abstract

**Figure:**
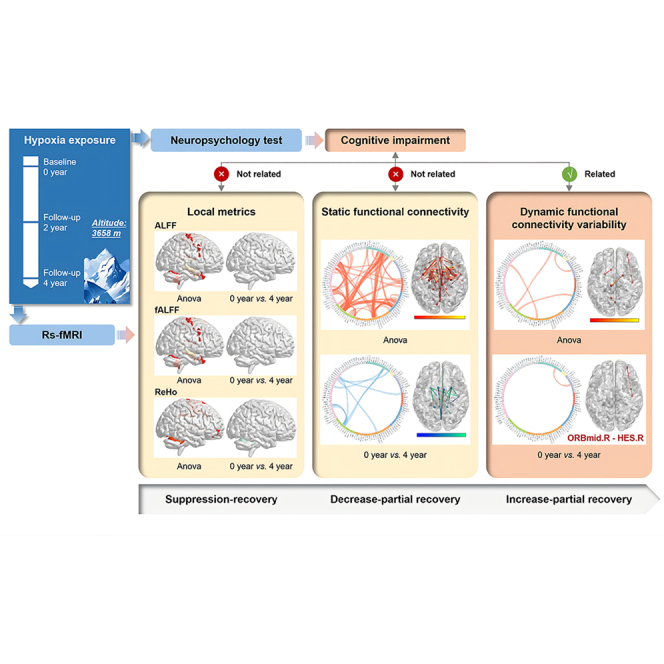

## Teaser

Health sciences; Cognitive neuroscience

## Introduction

With the expansion of tourism and economic development in high-altitude (HA) environments, more people are exposed to the negative health effects of HA conditions. Among the various environmental stressors at HA environments, hypoxia stands out as the most critical physiological challenge. The brain, as a high oxygen-consuming organ, is sensitive to alterations in oxygen supply. Although it represents approximately 2% of the total body mass, the brain consumes more than 20% of the body’s oxygen intake.^1^ Consequently, HA conditions can impose substantial strain on brain function. A growing body of research has demonstrated that exposure to HA environments can induce neuropsychological impairments, including deficits in memory, learning, and psychomotor performance.^2^^,^^3^^,^^4^ As a specific example, Wang et al. reported reduced accuracy and prolonged response times in the backward digit task under hypoxic conditions relative to normoxia.^3^ Additionally, Chen et al. found that chronic hypoxia exposure leads to significantly lower accuracy on memory tests as well as to longer reaction times.^2^ While multiple studies have attempted to characterize the physiological and neurological alterations that occur after ascent to HA environments, few studies have investigated the alterations that occur in cognitive and brain functions after long-term exposure to HA conditions.

As an advanced neuroimaging technique, resting-state functional magnetic resonance imaging (rs-fMRI) has been widely employed to identify differences in brain functions in patients with various neurological and cognitive disorders.^5^^,^^6^ rs-fMRI allows the measurement of spontaneous brain activity at rest via non-invasive detection of fluctuations in the blood-oxygen-level-dependent (BOLD) signal, which reflect hemodynamic changes associated with neuronal activity.^7^ Accordingly, rs-fMRI offers unique advantages in terms of both temporal and spatial resolution as well as good sensitivity to both cortical and subcortical neuronal activities. The rs-fMRI metrics most commonly used to assess the spontaneous activity characteristics of local brain regions include regional homogeneity (ReHo), amplitude of low-frequency fluctuations (ALFF), and fractional amplitude of low-frequency fluctuations (fALFF). ReHo reflects the local synchronization of neural activity by quantifying the temporal coherence of BOLD signals within a given voxel and its immediate neighbors.^8^ The ALFF is a measurement of the regional intensity of spontaneous oscillations within the low-frequency range and is calculated as the square root of the power spectrum integrated over this band.^9^ The fALFF provides refined specificity relative to the ALFF based on computation of the ratio of low-frequency power to the total frequency spectrum, which serves to mitigate contamination due to physiological noise.^9^ Previous studies have identified alterations in ReHo, ALFF, and fALFF in association with Alzheimer’s disease (AD) and suggested that these metrics may serve as biomarkers for AD diagnosis and therapeutic targeting.^10^^,^^11^ For example, Khatri and Kwon conducted a meta-analysis to investigate multimodal alterations in ALFF and fALFF in patients with AD or preclinical AD compared with healthy controls (HCs) and showed that patients with AD exhibit decreased ALFF/fALFF in the bilateral posterior cingulate gyrus (PCC), precuneus (PCUN), and right angular gyrus (ANG) as well as increased ALFF/fALFF in the bilateral parahippocampal gyrus (PHG) compared with HCs.^10^ We previously studied a group of college students who had immigrated to an HA plateau for 1 or 2 years; we observed significant decreases in ReHo in the left putamen (PUT), superior temporal gyrus (STG), superior parietal lobule (SPG), anterior cingulate gyrus (ACG), and medial frontal gyrus (MFG) along with an increase in ReHo in the hippocampus after exposure to HA conditions.^2^

The human brain exhibits remarkable structural and functional complexity, with cognitive processes emerging from the interplay of distinct neural substrates in different brain regions, which form interconnected networks to support cognitive function.^12^ Hence, evaluation of regional (local) metrics offers limited value in correlation research, as such metrics predominantly reflect the circumscribed neural activity within a specific brain region and cannot represent the network-level interactions essential for cognitive function. Accordingly, studies in the past decade have increasingly focused on the cooperation that occurs across large-scale brain networks.^13^ Interregional brain activity is now commonly assessed in terms of functional connectivity (FC), with different forms including static FC (sFC) and dynamic FC (dFC).^14^^,^^15^ sFC is determined by evaluating the temporal correlations in BOLD signals among distinct brain regions with the assumption that intrinsic activity remains stable over time, offering critical insight into normal and abnormal brain organization.^16^ In contrast, dFC captures time-varying interactions by characterizing spontaneous fluctuations in connectivity across sliding time windows, thereby enabling exploration of temporal integration among brain networks.^17^ Accumulating evidence indicates that combining sFC and dFC evaluations can provide a more comprehensive representation of neuropathological alterations than either approach alone.^18^^,^^19^ Hypoxia, as a diffuse and chronic stressor, exerts multifaceted effects on brain functions, and the resultant cognitive impairments arise from dysregulated interregional interactions across multiple brain regions rather than isolated damage to a single brain region. This systemic property underscores the utility of brain network analysis as a critical methodological framework for investigating hypoxia-induced neural alterations. Therefore, the present study involved combined sFC and dFC analyses to benefit from the advantages of both approaches.

Herein, we present the results of a 4-year-long longitudinal panel study conducted on a young healthy HA immigrant population to characterize the changes in cognition and other brain functions that occur with chronic HA exposure. This study tested our hypotheses that continuous alteration of brain functions would be observed over several years of chronic HA exposure and that these changes would contribute to decreased cognitive performance. To date, no longitudinal neuroimaging study has systematically investigated both static and dynamic FC alterations associated with chronic hypoxia-induced cognitive impairments.

## Results

### Establishment of the HA immigrant cohort

**Figure 1:**
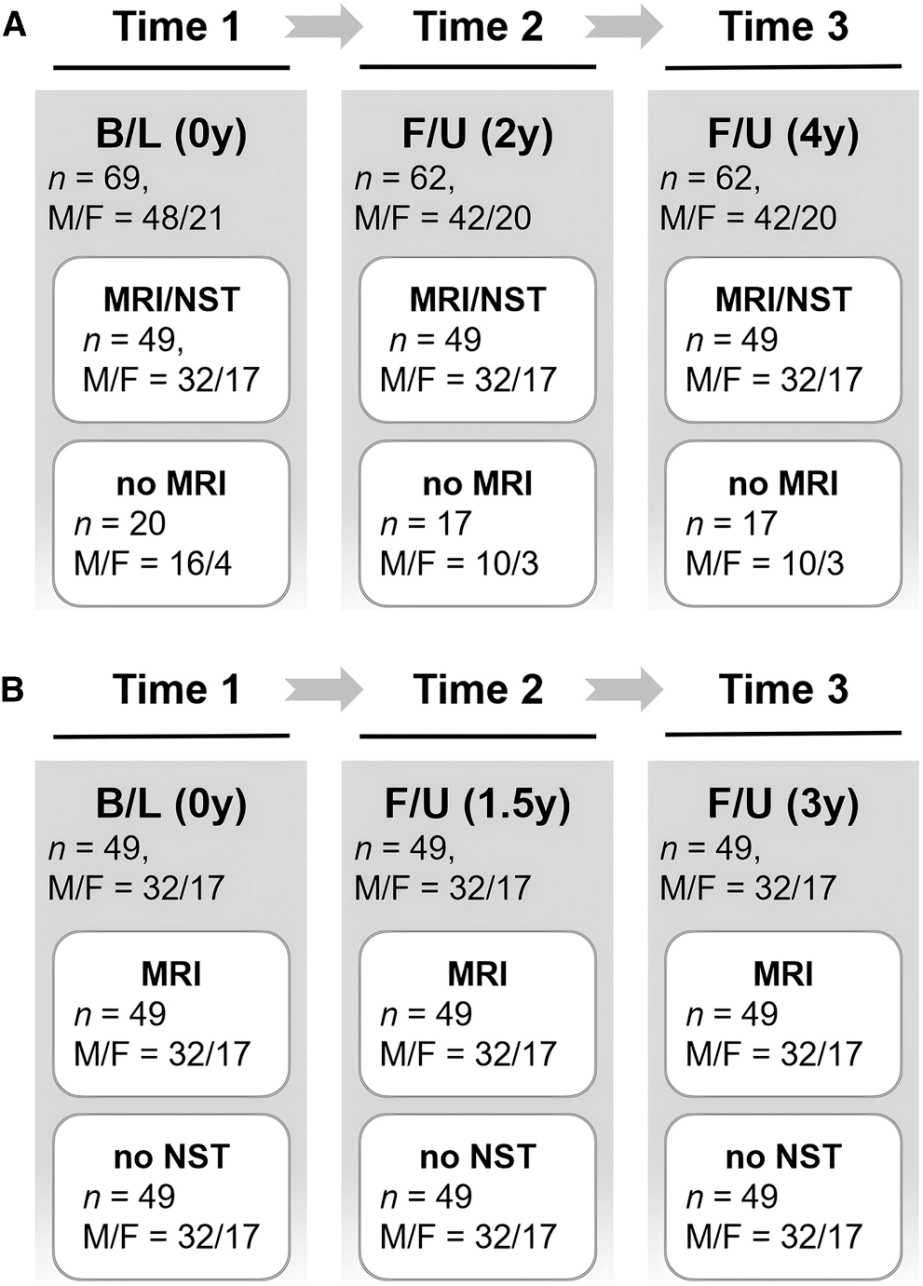
Flowchart of participant inclusion (A) Experimental sample. (B) Control sample. The study included experimental and control datasets. MRI, magnetic resonance imaging; NST, neuropsychology test.

Figure 1 presents a flow chart of participant recruitment and follow-up in this study. The present study enrolled young adults who immigrated to an HA region to receive higher education. Participants were followed for 4 years for the evaluation of HA-induced cognitive and brain functional network alterations. In the experimental group, the average cumulative HA exposure time from time 1 to time 2 was 560.8 ± 7.3 days (range: 541–573 days), and the average cumulative exposure time from time 2 to time 3 was 552.4 ± 7.5 days (range: 535–568 days). In the control group, the average number of days between the first and the second scanning sessions was 515 days and that between the second and the third scanning sessions was 520 days.

### HA exposure induced impairments in working memory and psychomotor function

**Figure 2:**
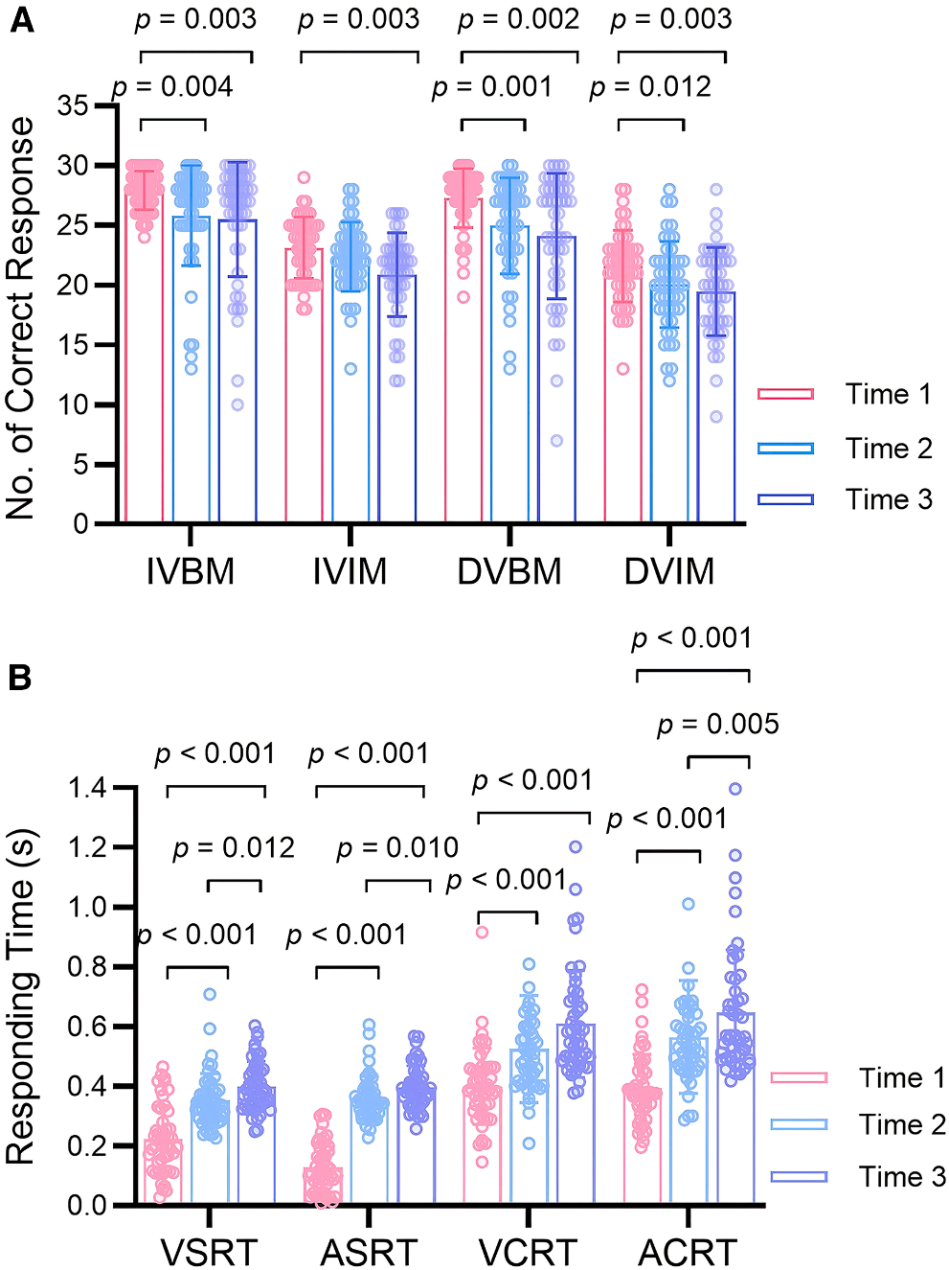
HA-exposure-induced impairments in working memory and psychomotor function (A) Number of correct responses for IVBM, IVIM, DVBM, and DVIM decreased significantly over the exposure time. (B) The participants’ VSRT, ASRT, VCRT, and ACRT were significantly prolonged after HA exposure. ACRT, auditory recognition reaction time; ASRT, auditory simple reaction time; DVBM, delayed verbal memory; DVIM, delayed visual memory; HA, high-altitude; IVBM, immediate verbal memory; IVIM, immediate visual memory; VCRT, visual recognition reaction time; VSRT, visual simple reaction time.

In this 4-year follow-up, we systematically evaluated HA-induced cognitive and neurobehavioral alterations by administration of neuropsychological tests to participants in the experimental group at baseline and follow-up examinations. The neuropsychological testing results revealed that HA exposure decreased accuracy on visual/auditory memory tests and prolonged response times in the visual/auditory reaction time tests (Table 1). Moreover, working memory performance declined significantly during the first 2 years of HA exposure and then remained at a reduced level thereafter, whereas reaction times progressively deteriorated throughout the study period (Figure 2). These observations indicate that HA-related hypoxia induced working memory and psychomotor impairments in the early stage of exposure, which may be irreversible with prolonged exposure time.

**Table 1:** Descriptive values and statistical analysis of altered cognitive performance metrics before and after high-altitude exposure (*n* = 49)

Cognitive performance metrics	Time 1 (mean ± SD)	Time 2 (mean ± SD)	Time 3 (mean ± SD)	*F*	*p*	ηp^2^	Post hoc comparison
IVBM, *n*	27.918 ± 1.631	25.837 ± 4.195	25.490 ± 4.801	5.643	0.005	0.105	time 1 > time 2 (*p* = 0.004)
							time 1 > time 3 (*p* = 0.003)
IVIM, *n*	23.122 ± 2.579	22.367 ± 2.885	20.878 ± 3.492	8.249	<0.001	0.147	time 1 > time 3 (*p* = 0.003)
DVBM, *n*	27.286 ± 2.475	24.959 ± 4.015	24.102 ± 5.245	8.073	0.001	0.144	time 1 > time 2 (*p* = 0.001)
							time 1 > time 3 (*p* = 0.002)
DVIM, *n*	21.592 ± 2.992	20.061 ± 3.602	19.469 ± 3.680	7.677	0.001	0.138	time 1 > time 2 (*p* = 0.012)
							time 1 > time 3 (*p* = 0.003)
VSRT, s	0.222 ± 0.113	0.352 ± 0.090	0.399 ± 0.084	62.939	<0.001	0.567	time 1 < time 2 (*p* < 0.001)
							time 1 < time 3 (*p* < 0.001)
							time 2 < time 3 (*p* = 0.012)
ASRT, s	0.128 ± 0.082	0.356 ± 0.076	0.395 ± 0.072	236.011	<0.001	0.831	time 1 < time 2 (*p* < 0.001)
							time 1 < time 3 (*p* < 0.001)
							time 2 < time 3 (*p* = 0.010)
VCRT, s	0.399 ± 0.128	0.524 ± 0.179	0.609 ± 0.179	22.237	<0.001	0.317	time 1 < time 2 (*p* < 0.001)
							time 1 < time 3 (*p* < 0.001)
ACRT, s	0.392 ± 0.114	0.564 ± 0.189	0.645 ± 0.211	57.054	<0.001	0.543	time 1 < time 2 (*p* < 0.001)
							time 1 < time 3 (*p* < 0.001)
							time 2 < time 3 (*p* = 0.005)

*n* represents the number of correct responses on memory tests; s represents the seconds of reaction time.

ACRT, auditory recognition reaction time; ASRT, auditory simple reaction time; DVBM, delayed verbal memory; DVIM, delayed visual memory; IVBM, immediate verbal memory; IVIM, immediate visual memory; VCRT, visual recognition reaction time; VSRT, visual simple reaction time.

### HA exposure induced alterations in physiological parameters

During the baseline and follow-up studies, we identified significant alterations in basic physical conditions. Specifically, red blood cell count (RBC), hemoglobin (HBG) concentration, heart rate (HR), respiratory rate (RR), systolic blood pressure (SBP), diastolic blood pressure (DBP), left ventricular ejection fractions (LVEFs), and left ventricular fractional shortening (LVFS) were elevated after HA exposure, indicating compensatory changes relative to baseline. Peripheral oxygen saturation (SpO2) displayed a notable decrease during the first 2 years and then stayed at the low level thereafter. Other physiological parameters, including vital capacity (VC), forced vital capacity (FVC), and forced expiratory volume in 1 s (FEV1), decreased gradually during the 4-year HA exposure period (Table S1).

### Local neural activity metrics exhibited a biphasic pattern characterized by initial suppression followed by full/partial recovery during the 4-year HA exposure period

**Figure 3:**
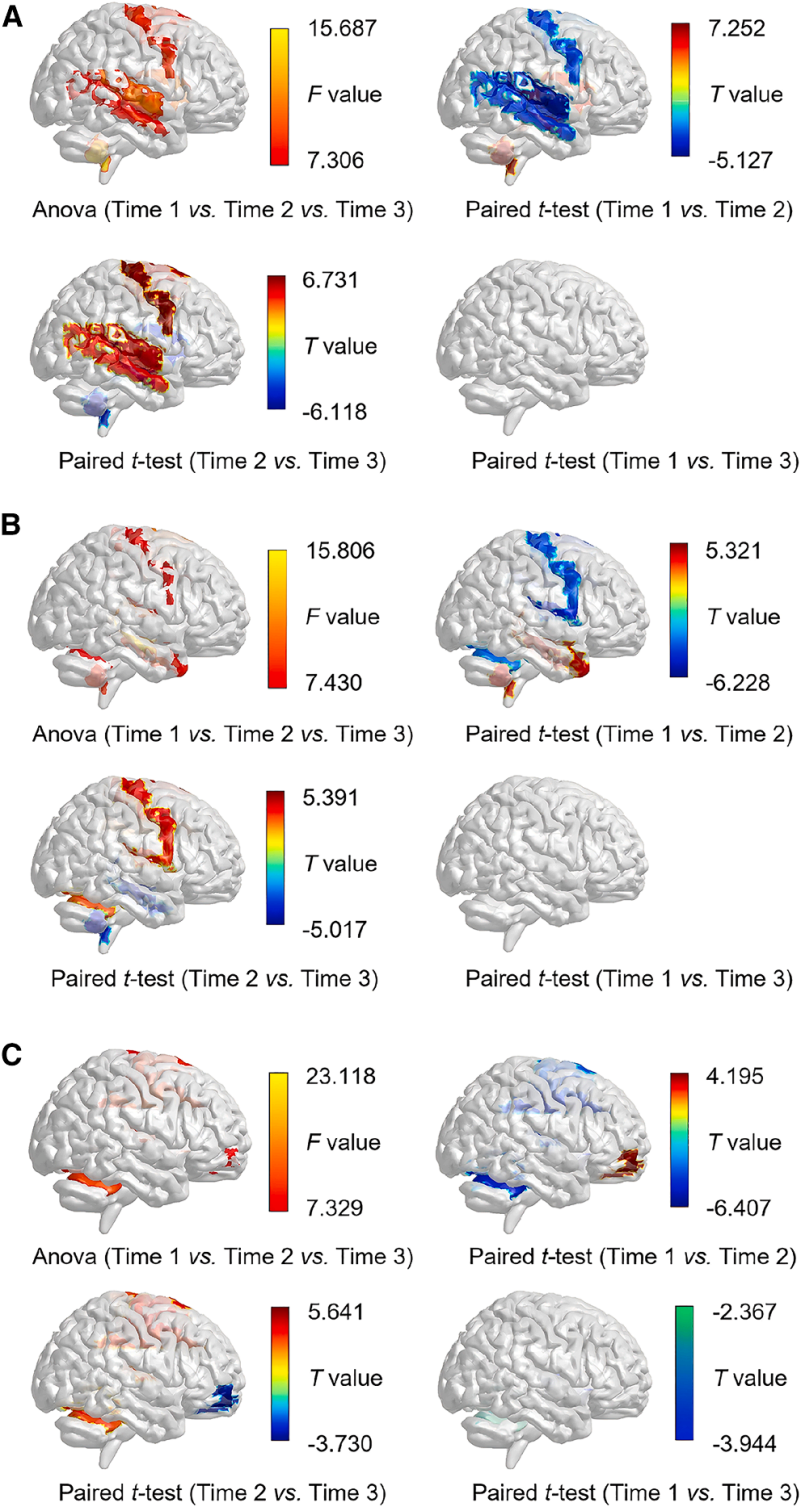
Local neural activity metrics exhibited a biphasic pattern characterized by initial suppression followed by full/partial recovery during the 4-year HA exposure period (A) ALFF values. (B) fALFF values. (C) ReHo values. Red areas indicate higher ALFF/fALFF/ReHo values, and blue areas indicate lower ALFF/fALFF/ReHo values in participants after HA exposure. ALFF, amplitude of low-frequency fluctuations; CRBL45.L, left cerebelum_4_5; CRBL6.L, left cerebelum_6; fALFF, fractional ALFF; HA, high-altitude; INS.L, left insula; PAL.L, left lenticular nucleus, pallidum; PUT.L, left lenticular nucleus, putamen; PUT.R, right lenticular nucleus, putamen; ReHo, regional homogeneity.

To explore the mechanism of chronic HA-exposure-induced neuropsychological impairment, we first conducted comparative analyses of local brain activity metrics, including ALFF, fALFF, and ReHo. In the experimental group, analysis of variance (ANOVA) of ALFF and fALFF results revealed significant differences within 15 and 14 brain regions, respectively. Notable regions included the somatomotor network (SMN), ventral attention network (VAN), limbic system (LS), basal ganglia (BG), and cerebellum (Cere) (all uncorrected *p* < 0.001; Figure 3; Tables S2 and S3). On post hoc multi-comparison, the ALFF and fALFF values for the aforementioned brain regions were significantly suppressed at time 2 but had fully recovered at time 3, with no significant differences in any region detected in comparisons of time 3 to time 1 values (all the false discovery rate [FDR]-corrected *p* < 0.05). Overall, the ALFF and fALFF values exhibited initial suppression followed by full recovery during the 4-year HA exposure period.

Similarly, ANOVA of ReHo revealed significant differences among the 18 brain regions, primarily in the SMN, VAN, BG, and Cere (uncorrected *p* < 0.001; Figure 3; Table S4). The ReHo values for the aforementioned brain regions were significantly suppressed at time 2 and had partially recovered at time 3. Additionally, brain regions, including the left insula (INS), left and right PUT, left pallidum (PAL), left cerebelum_4_5 (CRBL45), left cerebelum_6 (CRBL6), vermis_6 (Vermis6), and vermis_7 (Vermis7), showed significantly decreased ReHo values at time 3 compared with time 1 (all FDR-corrected *p* < 0.05). Overall, ReHo values exhibited initial suppression followed by partial recovery during the 4-year HA exposure period. Comparatively, no significant main effects were observed on the ANOVA of any local activity metric values in the control group (uncorrected *p* > 0.001). Moreover, after HA exposure, ANOVA identified significant gray matter volume (GMV) changes in the right PAL (GRF-corrected, voxel *p* < 0.001, cluster *p* < 0.01). Post hoc tests indicated its GMV dropped markedly at time 2 and stayed low at time 3 versus time 1, with no difference between time 2 and time 3 (Figure S1; Tables S13 and S14). Such alterations were not detected in control group (uncorrected *p* > 0.001).

Although the ReHo values for the INS, PUT, PAL, and Cere showed reductions during the 4-year HA exposure period, no notable associations were found between the changes in ReHo values and cognitive impairments (*p* > 0.05). Therefore, the present study suggests that behavioral changes in this population were not predominantly driven by changes in local activity metrics, and thus, the underlying mechanisms of cognitive alterations with HA exposure remained to be elucidated.

### sFC exhibited a dynamic pattern marked by initial reduction followed by partial recovery during the 4-year HA exposure period

**Figure 4:**
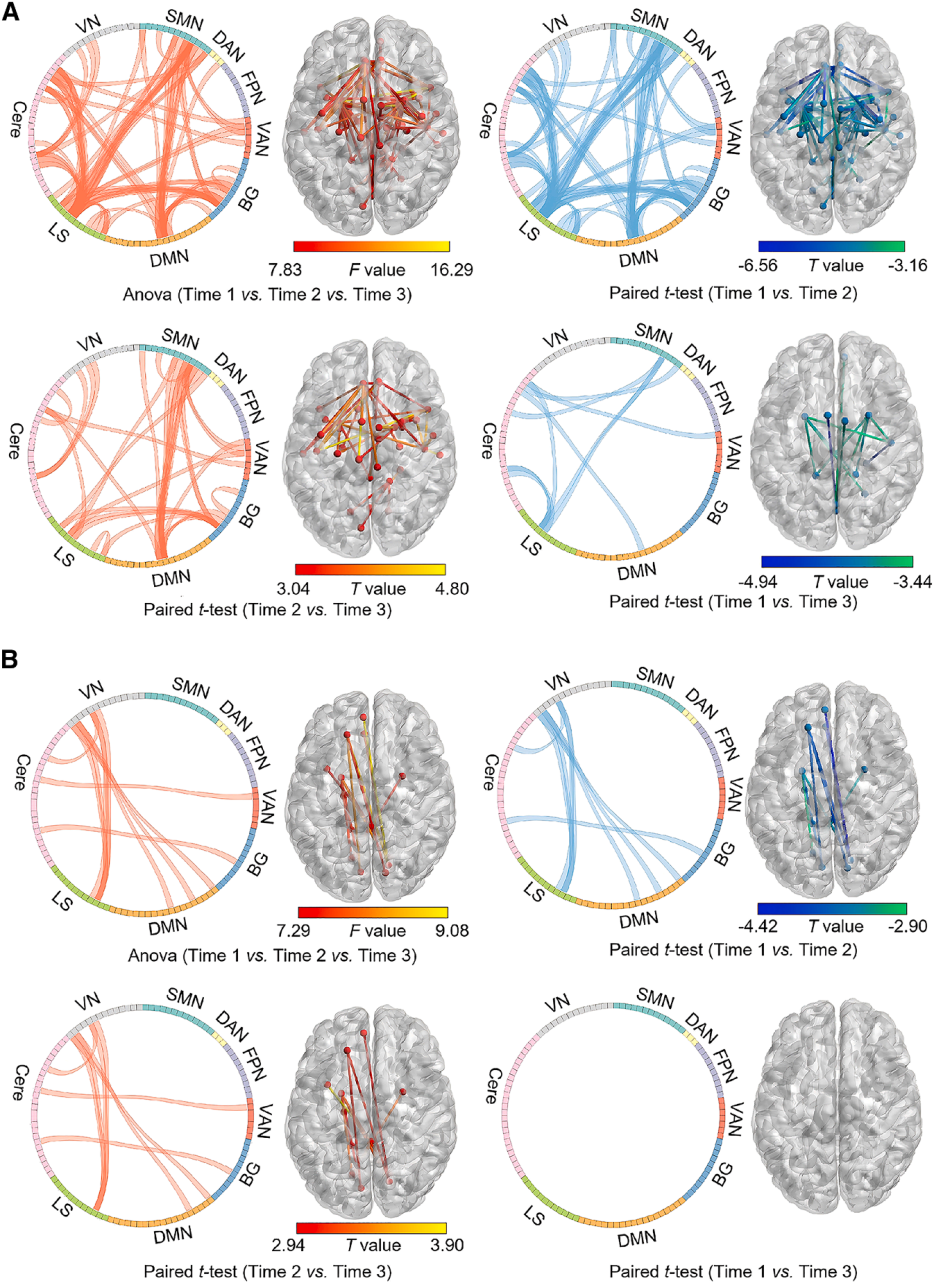
sFC exhibited a dynamic pattern marked by an initial reduction followed by partial recovery during the 4-year HA exposure period (A) In the experimental group, 12 edges involving the DMN, SMN, VAN, BG, and Cere were found to have significantly decreased sFC at time 3 compared with time 1. (B) In the control group, sFC exhibited an initial upward trend followed by full recovery during the study period at sea level. In the left circle maps, the surrounding colored squares represent different brain regions; the middle red strips represent the connected edges with significantly increased sFC after exposure; and the blue strips represent the connected edges with significantly decreased sFC after exposure. Maps on the right represent the distribution of brain sFC with differences before and after exposure from axial brain positions. BG, basal ganglia; Cere, cerebellum; DAN, dorsal attention network; DMN, default mode network; sFC, state functional connectivity; FPN, frontoparietal network; HA, high-altitude; LS, limbic system; SMN, somatomotor network; VAN, ventral attention network; VN, visual network.

To further investigate HA-exposure-induced brain functional alterations at a system-wide level, we examined changes in sFC in whole brain regions during the 4-year HA exposure period. In the experimental group, ANOVA revealed significant sFC differences in 80 of 13,456 edges (all uncorrected *p* < 0.001; Figure 4; Tables S5, S6, S7, S8). Among these edges, sFC values for 79 edges were decreased at time 2, and the values for 36 of these edges had recovered at time 3. Twelve edges involving the default mode network (DMN), SMN, VAN, BG, and Cere were found to have significantly decreased sFC values at time 3 compared with time 1 (all FDR-corrected *p* < 0.05), indicating that the sFC reductions for these edges persisted during the exposure period. To verify whether the sFC alterations for these edges contribute to HA-induced cognitive impairments, we conducted correlation analyses between the sFC alterations and cognitive changes from before to after HA exposure. However, this analysis showed no associations between the sFC alterations and the changes in cognitive performance (*p* > 0.05).

In the control group, ANOVA identified 11 edges with significant changes in sFC (uncorrected *p* < 0.001), but no alterations in sFC values were observed for any edge when comparing time 1 values to time 3 values (Figure 4; Tables S9 and S10; all FDR-corrected *p* < 0.05).

Consistent with the results for ReHo, sFC exhibited a dynamic pattern marked by an initial reduction followed by partial recovery. Several edges showed persistent sFC alterations after chronic hypoxia exposure, especially in the motor-related brain regions, including the PUT, Heschl’s gyrus (HES), and Cere. Although these changes were not associated with cognitive performance, we still hypothesize that FC plays a vital role in maintaining the stability and adaptability of cognitive function through the adjustment and optimization of neural network activities. Therefore, the present study further investigated whether FC related to brain functional variability in the time dimension (dFC) could offer more insight into the mechanism of cognitive changes than the mean brain FC (sFC).

### dFC variability in right ORBmid-right HES increased with chronic HA exposure and correlated significantly with cognitive impairments

**Figure 5:**
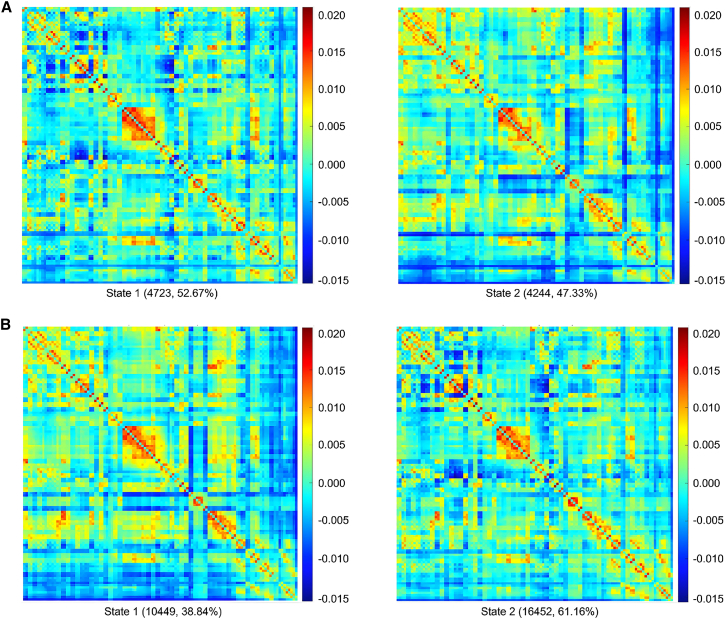
Cluster centroids for each state across all participants with the number of occurrences and percentage of occurrences listed above the autocorrelation matrix (A) In the experimental group, state 1: frequency 52.67%; state 2: frequency 47.33%. (B) In the control group, state 1: frequency 38.84%; state 2: frequency 61.16%.

Due to the limitations of sFC, which cannot reflect the time-varying features of the complex neural system of the brain, we further explored the alterations in FC from a dynamic perspective. In the experimental group, through the sliding window, elbow criterion, and *k*-means clustering analyses, two states of dFC were obtained, for which the centroids are displayed in Figure 5. The proportions of the two states at all time points for all participants were 52.67% and 47.33%. Moreover, in the two states, ANOVA results for dFC temporal properties showed no significant main effects of HA exposure on the number of transitions (NT), the mean dwell time (MDT), fractional time (FT), or strength of dFC network (SDFCN) (uncorrected *p* > 0.001).

**Figure 6:**
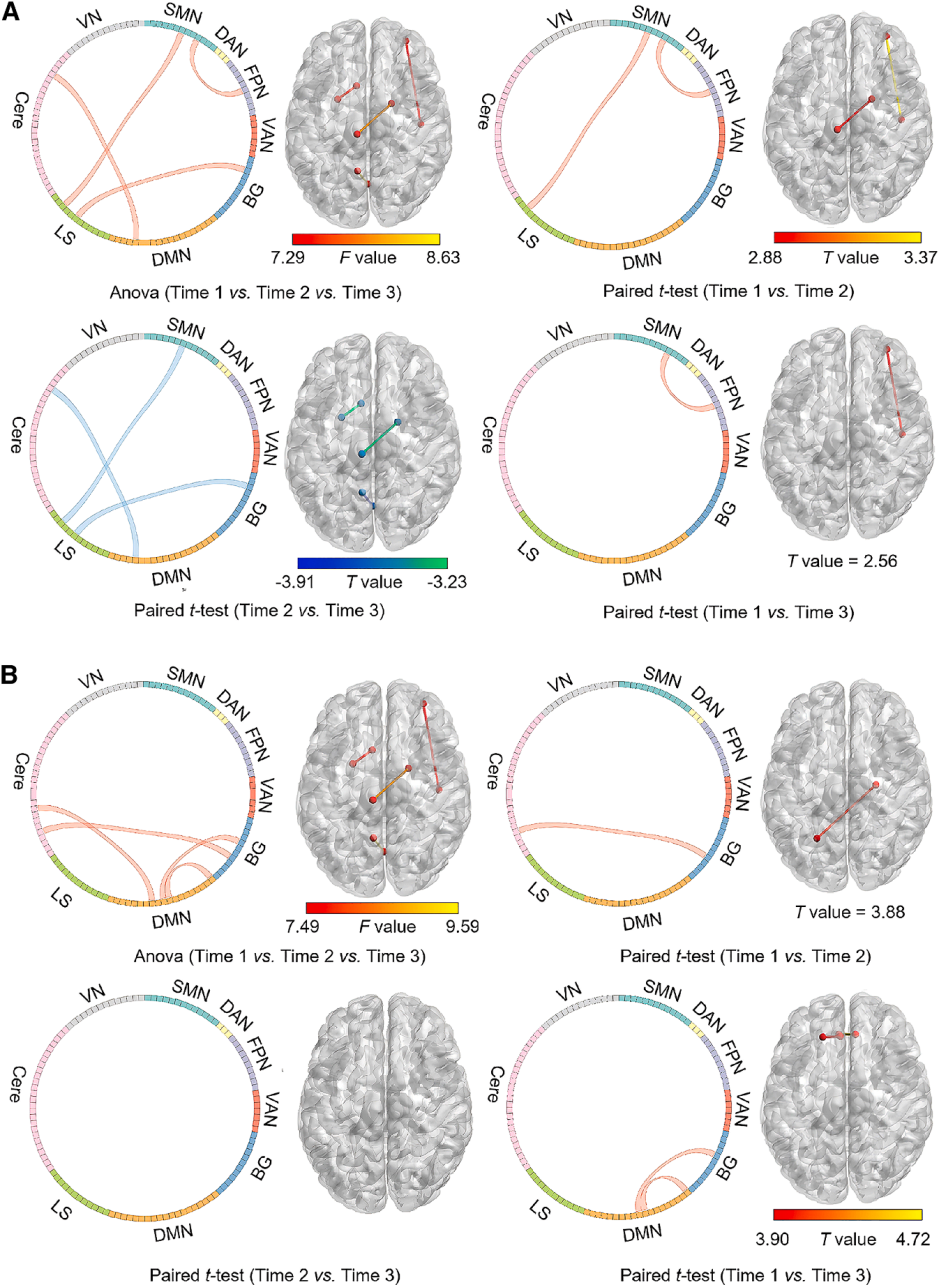
dFC variability exhibited an initial upward trend with recovery for a majority of edges during the 4-year HA exposure period (A) In the experimental group, the edge of dFC variability between the ORBmid.R and HES.R within the FPN-SMN remained elevated at time 3 compared with time 1. (B) In the control group, dFC variability exhibited an initial upward trend and fully recovered over the study period at sea level. In the left circle maps, the surrounding colored squares represent different brain regions; the middle red strips represent the connected edges with significantly increased dFC variability after exposure; and the blue strips represent the connected edges with significantly decreased dFC variability after exposure. BG, basal ganglia; Cere, cerebellum; DAN, dorsal attention network; dFC, dynamic functional connectivity; DMN, default mode network; FPN, frontoparietal network; HA, high-altitude; LS, limbic system; SMN, somatomotor network; VAN, ventral attention network; VN, visual network.

In the analysis of dFC variability in the experimental group, ANOVA revealed significant differences in dFC variability within 4 edges, including the left olfactory cortex (OLF)-left PUT, left paracentral lobule (PCL)-right PAL, right orbital middle frontal gyrus (ORBmid)-right HES, and left PCUN-Vermis6 (uncorrected *p* < 0.001; Figure 6; Tables 2 and 3). Among these edges, the right ORBmid-right HES and left PCL-right PAL edges showed significantly elevated dFC variability at time 2, indicating that the fluctuations of FC in the two edges became unstable after 2 years of HA exposure. Moreover, the dFC variability in the right ORBmid-right HES edge remained elevated at time 3 compared with time 1, demonstrating persistent dysregulation of connectivity fluctuations induced by chronic HA exposure. In contrast, the dFC variability in the left PCL-right PAL edge had recovered at time 3, which may represent an adaptation to chronic HA exposure. Additionally, the dFC variabilities in left OLF-left PUT and left PCUN-Vermis6 edges at time 3 were significantly lower than those at time 2 but did not differ significantly from those at time 1 (all FDR-corrected *p* < 0.05), which may indicate a potential compensatory effect of brain networks under chronic HA exposure.

**Table 2:** One-way repeated measures ANOVA of alterations in dFC variance before and after high-altitude exposure (*n* = 49)

Brain regions	Z1	Z2	Z3	*F*	*p*	ηp^2^
OLF.L-PUT.L	0.012	0.022	0.012	7.349	0.001	0.093
PCL.L-PAL.R	0.017	0.030	0.015	8.016	0.001	0.100
ORBmid.R-HES.R	0.012	0.026	0.018	7.285	0.001	0.092
PCUN.L-Vermis6	0.017	0.026	0.009	8.626	< 0.001	0.107

Uncorrected *p* < 0.001. Z1, Z2, and Z3 indicate the mean values of dFC variance before and after high-altitude exposure at time 1, time 2, and time 3, respectively.

dFC, dynamic functional connectivity; OLF.L, left olfactory cortex; ORBmid.R, right middle frontal gyrus, orbital part; PCL.L, left paracentral lobule; PCUN.L, left precuneus; PUT.L, left lenticular nucleus, putamen; PAL.R, right lenticular nucleus, pallidum; HES.R, right Heschl’s gyrus.

**Table 3:** One-way repeated measures ANOVA of alterations in dFC variance with paired *t* test Results before and after high-altitude exposure (*n* = 49)

Time 1 vs. Time 2			Time 2 vs. Time 3			Time 1 vs. Time 3		
Brain regions	*t*	*p*	Brain regions	*t*	*p*	Brain regions	*t*	*p*
PCL.L-PAL.R	2.875	0.006	OLF.L-PUT.L	−3.233	0.002	ORBmid.R-HES.R	2.560	0.014
ORBmid.R-HES.R	3.374	0.001	PCL.L-PAL.R	−3.285	0.002	–	–	–
–	–	–	PCUN.L-Vermis6	−3.908	<0.001	–	–	–

FDR-corrected *p* < 0.05.

dFC, dynamic functional connectivity; HES.R, right Heschl’s gyrus; PAL.R, right lenticular nucleus, pallidum; PCL.L, left paracentral lobule; ORBmid.R, right middle frontal gyrus, orbital part.

**Figure 7:**
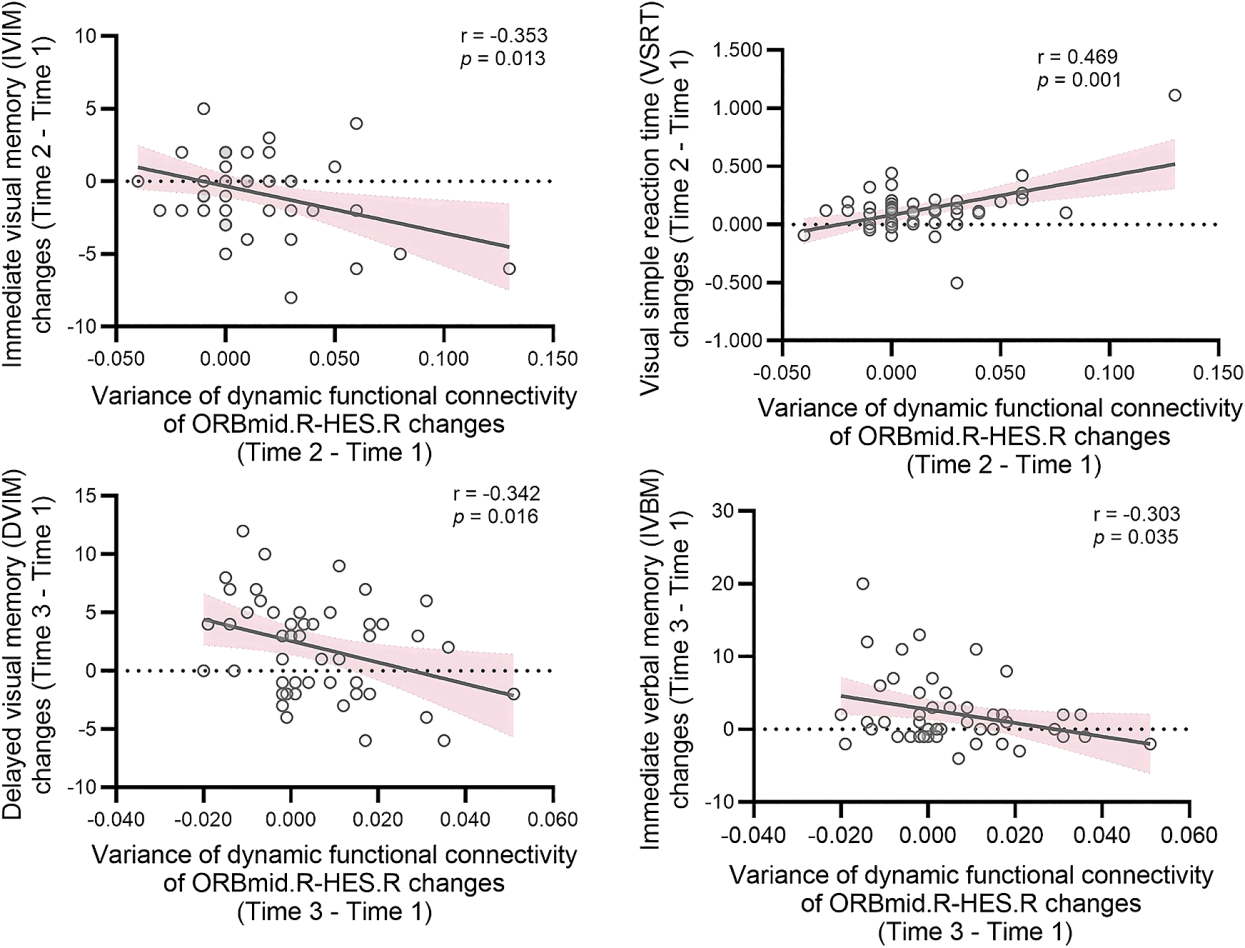
Correlations between dFC variability changes and cognitive impairments in the experimental group The dFC variability changes between the right ORBmid and right HES from time 2 to time 1 correlated negatively with the accuracy changes for IVIM and corrected positively with the VSRT after 2 years of HA exposure. Additionally, the dFC variability changes between the right ORBmid and right HES correlated negatively with the accuracy changes for DVIM and IVBM from time 3 to time 1.

Correlation analyses revealed significant relationships between changes in dFC variability and cognitive impairments in the experimental group. Specifically, we observed a positive relationship between right ORBmid-right HES dFC changes and the changes in visual simple reaction time (VSRT) (r = 0.469, *p* < 0.01). Additionally, we observed negative correlations between right ORBmid-right HES dFC changes and diminished accuracy for immediate visual memory (IVIM) (r = −0.353, *p* < 0.05), delayed visual memory (DVIM) (r = −0.342, *p* < 0.05), and immediate verbal memory (IVBM) (r = −0.303, *p* < 0.05; Figure 7).

In the control group, two dFC states were identified (Figure 5). ANOVA of the dFC temporal properties showed no significant main influences on NT, MDT, FT, and SDFCN in the two states (uncorrected *p* values > 0.001). ANOVA also revealed a total of 5 edges with significant changes in dFC variability, but no significant differences in dFC were detected from time 3 to time 2 (Figure 6; Tables S11 and S12; all FDR-corrected *p* < 0.05).

Overall, dFC variability in the right ORBmid-right HES edge was found to be elevated and remained high with prolonged HA exposure. Notably, the alteration of dFC variability was significantly associated with cognitive changes, indicating that the alteration of the right ORBmid-right HES dFC variability may be the functional basis of HA-exposure-induced cognitive impairments.

## Discussion

With the continued socioeconomic development in HA areas, more people are choosing to immigrate there in pursuit of better economic prospects, cultural experiences, and quality of life. However, chronic exposure to hypoxia at HA areas can have significant negative effects on both the physical and cognitive functions of human, which, in turn, restrict work efficiency. Hence, HA-induced cognitive impairments represent an important public health problem in HA regions requiring urgent solutions. Previous studies have predominantly examined the adverse neural effects of acute HA exposure,^2^^,^^20^^,^^21^ and longitudinal studies exploring the effects of prolonged hypoxia on the brain remain scarce. Such studies that have been reported are commonly limited by an insufficient follow-up duration due to time and economic constraints. To overcome these limitations, the present study established a longitudinal cohort of Chinese immigrants to an HA area and employed neurobehavioral tests and rs-fMRI technology to identify brain functional alterations systematically based on local metrics (ReHo, ALFF, and fALFF) and by comparing sFC and dFC before and after HA exposure. Through this comprehensive framework, we aimed to elucidate the brain functional basis of potential cognitive impairments that occur with chronic HA exposure.

The results of the present study indicate that HA exposure decreased accuracy in working memory tests and prolonged response times in the reaction time tests, confirming that chronic HA exposure can induce working memory and psychomotor impairments, consistent with previous findings.^2^^,^^4^^,^^22^ A previous meta-analysis reported that psychomotor function and long-term memory show pronounced declines, while working memory and language skills show moderate decreases due to HA exposure.^23^ Notably, neither the accuracy of working memory nor the response time recovered over time at HA exposure, suggesting that chronic HA exposure may lead to persistent cognitive impairments. Interestingly, working memory performance declined significantly during the initial exposure period (before year 2) and remained at a reduced level thereafter, whereas reaction times exhibited progressive deterioration. These findings indicate that psychomotor function may be more vulnerable to HA-induced deficits compared with working memory, highlighting the need for targeted interventions to mitigate these specific impairments. A large community-based study has characterized normative lifespan trajectories of inhibitory control, working memory, cognitive flexibility, and planning ability in healthy individuals spanning 10–86 years of age.^24^ The results showed that working memory capacity and planning ability continue to develop over adolescence and into early adulthood.^24^ Collectively, these findings indicate that chronic HA exposure exerts a significant and persistent adverse effect on the cognitive function of healthy young adults, even during a key developmental stage in which their core cognitive capacities are still undergoing active maturation.

During the 4 years of HA exposure, ALFF/fALFF values showed initial suppression followed by full recovery. ALFF reflects the spontaneous neural activity within a brain region based on quantification of the power of low-frequency BOLD signal fluctuations, whereas fALFF provides a more specific measure of spontaneous neural activity and further scales the average ALFF signal to the average amplitude across the whole low-frequency range to remove white and physiological noise.^9^ In the early stage of HA exposure, we observed significantly reduced ALFF/fALFF values in several brain regions, suggesting diminished spontaneous neural activity. Previous studies have demonstrated that such reductions may be caused by a decrease in oxygen partial pressure, metabolic disturbances, or impaired neural synchronization under hypoxic conditions.^9^^,^^21^ In the early stage of HA exposure, we observed significantly reduced ALFF/fALFF values across key brain networks, including the SMN, VAN, and DMN. These networks underlie sensorimotor integration, salience and attentional processing, and self-referential cognitive regulation, respectively.^25^ Such widespread reductions in intrinsic neural activity suggest that hypoxia-induced metabolic constraints dampen baseline neural dynamics, thereby disrupting integrated cognitive function. These findings align with existing studies reporting hypoxia-related functional suppression and may contribute to the cognitive alterations associated with prolonged HA exposure.^9^^,^^21^ Interestingly, our study also found that the ALFF/fALFF values of a small number of brain regions within the LS, BG, and Cere were relatively increased after 2 years of HA exposure compared with baseline values, which suggests a selective enhancement of spontaneous neuronal activity in these regions with chronic hypoxia exposure. We propose that the vulnerability to hypoxia and responsiveness to hypoxia tolerance differed among these regions due to variations in the local blood supply to these brain regions. The LS modulates emotional and stress responses, the BG regulates motor control and reward processing, and the Cere coordinates motor execution and cognitive integration. This selective enhancement of neural activity may reflect a neuroprotective mechanism to preserve core affective, motor, and regulatory functions under prolonged hypoxic exposure.^26^^,^^27^ In the later stage of HA exposure (2–4 years), we found that the ALFF/fALFF values had fully recovered to baseline levels. This restoration likely reflects two compensatory mechanisms: (1) neuroplastic adaptation, particularly synaptic plasticity facilitating the reinstatement of regional spontaneous activity and inter-network functional coherence and (2) metabolic compensation through functional remapping and recruitment of alternative neural pathways to offset initial hypofunction.^4^ The biphasic response pattern of these metrics demonstrates the remarkable functional plasticity of the brain for adapting to chronic environmental stressors, specifically sustained hypobaric hypoxia in our experimental population.

During the 4-year study period of HA exposure, ReHo and sFC exhibited initial reductions followed by partial recovery, especially in motor-related brain regions, including the PUT, HES, and Cere, which showed persistent alterations before and after chronic hypoxia exposure. ReHo provides a measure of the local synchronization of spontaneous neural activity and a metric for assessing the similarity (or coherence) of the BOLD signal time series between a specific voxel and its neighboring voxels.^8^ FC refers to the temporal correlation of neural activity between spatially distinct brain regions.^16^ Specifically, the observed decreases in ReHo and sFC during the initial 2 years of HA exposure reflected the brain’s response to the acute stress caused by hypoxia, characterized by disrupted neural synchronization and reduced inter-regional coordination.^2^ These reductions in the first 2 years may stem from hypoxia-induced metabolic stress, altered neurovascular coupling, or transient neuroplasticity aimed at stabilizing neural activity under oxygen scarcity. The subsequent partial recovery of ReHo and sFC values after 2 years suggests the effects of adaptive mechanisms, such as enhanced erythropoiesis, angiogenesis, increased cerebral blood flow, or compensatory neuroplasticity, which mitigate hypoxia-induced damage.^28^ However, the brain regions that exhibited persistent reductions in ReHo and sFC were predominantly localized to motor-related areas (PUT, HES, and Cere). Given the high metabolic demands of these regions, they may experience cumulative oxidative stress under hypoxic conditions, rendering them particularly susceptible to impaired functional recovery.^9^ The Cere serves a crucial role in motor coordination. Similarly, the PUT contributes critically to motor regulation through its coordinated function with other structures in modulating voluntary movement initiation, execution, and inhibition.^27^^,^^29^ These specialized motor functions may increase susceptibility to non-motor functional impairments during adaptation, potentially due to insufficient compensatory neuroplastic mechanisms. Moreover, consistent with our previous findings, chronic HA exposure was found to significantly reduce both ReHo in the PUT and sFC between the PUT and other regions.^2^ These concurrent reductions in ReHo and sFC suggest that hypoxia may impair interregional functional integration by disrupting local neuronal activity within a single brain region. Notably, paralleling ReHo reductions in the BG, we also observed a significant GMV decrease in the right PAL, supporting the presence of concurrent structural and functional alterations in motor-related subcortical regions under chronic hypoxia, and underscore the importance of targeting subcortical motor circuits in future research on hypoxia-associated brain changes.

The present study delineated two distinct states through the clustering analysis. Specifically, state 1 represents a globally integrated state with enhanced inter-network functional coupling across large-scale brain systems, supporting efficient cross-network information integration critical for coordinating sensory processing and higher-order cognitive control. State 2 is a network-segregated state characterized by weakened inter-network connectivity and strengthened local modular processing, which sustains basic sensory-motor functions but limits complex cross-network cognitive operations. Notably, the hypoxia-exposed experimental group was dominated by the integrated state 1 (52.67%), whereas the control group showed a predominance of the segregated state 2 (61.16%). This striking shift in state fractional occupancy indicates that chronic HA-induced hypoxia drives a compensatory elevation in integrated dFC state occupancy, reflecting the brain’s core adaptive strategy to preserve inter-network communication. In addition, dFC analysis offers enhanced characterization of functional malfunctions through the capture of time-varying continuous features on short time scales.^15^ In the present study, dFC variability first increased with HA exposure and then returned to normal levels during the 4-year HA exposure period. However, the increasing trend of right ORBmid-right HES dFC was maintained with chronic HA exposure. As a component of the orbitofrontal cortex and part of the frontoparietal network (FPN), the ORBmid plays a crucial role in high-order cognitive processes.^30^ The HES, which is in the superior temporal plane, contributes to the SMN through its involvement in auditory processing, prosody perception, attentional modulation, and social-emotional cognitive functions.^31^ Despite limited evidence for a direct anatomical pathway between the ORBmid and the HES, FC between these two regions might be more likely to occur indirectly through intermediary regions (such as other parts of the superior or middle temporal lobe, the INS, or via multimodal association cortices). As a functional hub globally, the FPN exhibits a high degree of FC as well as a large degree of connectivity to many diverse brain networks.^32^^,^^33^ Hence, the ORBmid might interact with auditory regions like the HES via higher-order association networks that integrate auditory information with cognitive and emotional processing. Moreover, the SMN is a critical region for verbal short-term memory in coordination with the fronto-temporal areas.^33^^,^^34^ In patients with Parkinson’s disease, the SMN is associated with the fronto-executive dysfunction cognitive profile, which mainly impacts the FPN.^35^ Increased dFC variability between the right ORBmid and right HES may reflect unstable temporal coordination between auditory processing and cognitive control networks under chronic hypoxia. This pattern could represent either a compensatory adaptation to hypoxic stress or progressive network instability driven by impaired neurovascular coupling, metabolic mismatch, and disrupted synaptic transmission. Together, these alterations might compromise the integration of sensory input and executive control.

Notably, the alteration of dFC variability was significantly associated with cognitive changes. The association between increased right ORBmid-right HES dFC variability and reduced cognitive performance suggests that HA-related fluctuations in functional connectivity may disrupt the integration of auditory processing and higher-order cognitive control. Mechanistically, hypoxia disturbs the neuronal microenvironment, suppresses synaptic transmission, and reduces neural activity.^21^ Chronic hypoxia also impairs cerebrovascular autoregulation, while abnormal frontal activation increases metabolic and cerebral blood flow demand.^36^^,^^37^ The resulting mismatch between oxygen supply and demand disrupts neurovascular coupling, which may further aggravate local hypoxia and contribute to cognitive decline.^3^ This observed variability could represent either an adaptive response or a compensatory mechanism, suggesting an exciting new brain target linking aberrant dFC variability to chronic hypoxia-induced cognitive impairments. Compared with sFC metrics, which reflect only time-averaged interaction strength, dFC measures are capable of capturing moment-to-moment fluctuations in network configuration, which may render them more sensitive to subtle, early stage cognitive alterations that are not detectable by static indices. Thus, the observed elevation in dFC variability may represent a sensitive neural signature of disrupted cross-network integration under chronic hypoxic stress. Clinically, this staged pattern of temporary neural hypoactivity and incomplete functional recovery delivers quantitative neuroimaging proof describing brain adaptive remodeling triggered by long-term exposure.^38^ These results highlight the translational value of dFC variability as a standardized imaging readout for regular population brain health screening among hypoxia-exposed populations.

In the present study, we conducted a 4-year-long longitudinal panel study on a young healthy HA immigrant population with the objective of investigating how brain functional alterations are associated with the cognitive changes related to chronic HA exposure. Multiple cognitive functions, including working memory and psychomotor function, appeared to be affected following prolonged HA exposure. Specifically, the ALFF/fALFF values exhibited initial decreases followed by full recovery, whereas ReHo and sFC decreased initially and then partially recovered during the 4-year HA exposure period. Additionally, an initial increasing trend in dFC variability was observed, with later recovery occurring between some brain regions. However, the increased dFC variability between the right ORBmid and right HES was significantly associated with the cognitive changes that occurred over the 4-year HA exposure period, providing insight into a possible mechanism underlying the HA-induced alterations in cognitive function. Our findings highlight dFC variability as a potential biomarker for monitoring brain health with exposure to chronic hypoxia, which may guide targeted interventions for HA residents or workers. In future studies, prospective designs with classification and predictive modeling will be valuable to further distinguish different exposure time points and predict cognitive changes, thereby highlighting the discriminative and translational potential of dFC variability.

### Limitations of the study

Several limitations of this study should be considered. First, the sample size was small, which may have led us to miss potential findings. Accordingly, further studies with larger sample sizes and various task contexts are needed. Second, we did not assess longitudinal changes in cognition domains among the healthy controls living at sea level, which limits the generalizability of our findings. Another limitation is the difference in scanner types used between the HA and control groups, which may introduce subtle scanner-related variability despite preprocessing efforts. Specifically, scanner variability can affect key imaging metrics relevant to the present study, including the signal-to-noise ratio, BOLD signal amplitude and temporal dynamics, and temporal noise characteristics. These differences may further influence the accurate estimation of sFC and dFC, potentially compromising the comparability of imaging results between the HA and the control groups. Although we performed standardized preprocessing procedures to minimize such effects, residual scanner-related variability cannot be completely excluded. Future studies should adopt consistent scanner equipment or implement data harmonization or sensitivity analyses across groups, which will help mitigate this limitation and improve the robustness of findings.

## Resource availability

### Lead contact

Requests for further information and resources should be directed to and will be fulfilled by the lead contact, Xiaoming Chen (xiaomingchen@fmmu.edu.cn).

### Materials availability

This study did not generate new materials.

### Data and code availability

• All data reported in this paper will be made available from the lead contact upon request.
• The code reported in this paper will be made available from the lead contact upon request.
• Any additional information required to reanalyze the data reported in this paper is available from the lead contact upon reasonable request.

## Acknowledgments

We are grateful to all the participants in the study. This work was financially supported by the 10.13039/100014718National Natural Science Foundation of China (grant nos. 82271920 and 82230063), 10.13039/501100002858China Postdoctoral Science Foundation General Fund (grant no. 2024M764331), and 10.13039/501100009558Natural Science Research Project of Shaanxi Province (grant no. 2025JC-YBQN-1079).

## Author contributions

S.Z., S.G., Y.Z., B.L., J.L., W.L., and X.C. developed the study concept and design; J.H., Y.O., T.Y., J.W., and S.Z. carried out data collection; S.Z. performed the data analysis; S.Z., S.G., and X.C. completed the manuscript. All authors approved the final version of the paper for submission.

## Declaration of interests

Authors declare that they have no conflict of interest.

## STAR★Methods

### Key resources table

**Table:** 

REAGENT or RESOURCE	SOURCE	IDENTIFIER
**Software and algorithms**		

MATLAB	https://www.mathworks.com/	R2023a
RESTplus toolbox	http://www.restfmri.net/forum/	1.25 version
DynamicBC toolbox	http://restfmri.net/forum/DynamicBC/	2.0 software
GRETNA toolbox	http://www.nitrc.org/projects/gretna/	2.0 software
IBM SPSS	https://www.ibm.com/products/spss-statistics	29.0 software

### Experimental model and study participants details

#### Study design

For this study, all participants in the experimental group were from the Shaanxi–Tibet immigrant cohort and were admitted to Tibet University in Lhasa (average altitude: 3,658 m), China for a 4-year higher education period. Participants had completed a series of questionnaires covering HA exposure history, medical history, academic performance, and sociodemographic information, including parental education, vocation, and socioeconomic status. Figure 1 provides an overview of the study design. Data collection began in July 2014, and follow-up investigations including neuropsychological assessments were performed in June 2015, May 2016, and May 2018. MRI data were collected at three time points in 2014, 2016 and 2018. During follow-up assessments, participants were asked to provide information about the duration of HA exposure, their experience in mountain climbing, any low-altitude visits, and any medical events that occurred during the study period. At the same time, some physiological parameters were collected including RBC, HBG, HR, RR, SBP, DBP, LVEF, LVFS, SpO2, VC, FVC, and FEV1. The protocol for this study was approved by the Ethics Committee of the Medical Faculty of Fourth Military Medical University (registry No. KY20143344-1), and the study adhered to the ethical principles for medical research as stated in the Declaration of Helsinki.

The control group consisted of healthy college students for whom longitudinal MRI data were available for download from the open access Southwest University longitudinal imaging multimodal (SLIM) Brain Data Repository. This use of data from the SLIM database was approved by the Research Ethics Committee of the Brain Imaging Center of Southwest University. The control cases were selected based on gender and age matching with the participants in the experimental group.

#### Participants

For this study, we screened a total of 69 healthy right-handed high school graduates of the Shaanxi–Tibet immigrant cohort (18.62 ± 0.99 years, 37.68% female). Participants were enrolled in the study if they met the following inclusion criteria: (1) age 17–20 years; (2) altitude of permanent residence is less than 900 m; (3) no high-altitude exposure (≥2500 m) in the past; (4) no history of smoking, alcohol, and drug abuse; (5) no history of medications that may affect cognitive function or carbonic anhydrase activity; and (6) no chronic or genetic diseases. All enrolled participants in this study signed the informed consent form prior to the experiment on the premise of fully understanding the content of the experiment. Twenty of these students were excluded from the present study due to incomplete MRI data or questionnaire responses. The final experimental group included 49 students with a mean age of 18.73 ± 0.84 years and female proportion of 34.69%. A full description of this cohort was published with our previous study.^2^

The control group also included 49 healthy college students (19.31 ± 0.71 years, 34.69% female) from the SLIM Brain Data Repository. Control participants were screened for eligibility based on an age of 17–20 years, status as university freshmen or sophomores and fluency in Chinese. Control participants were excluded if any of the following criteria were met: (1) MRI-related exclusion criteria, such as claustrophobia, metallic implant, Meniere’s syndrome, or a history of fainting within the previous 6 months; (2) current psychiatric or neurological disorder; (3) use of psychiatric drugs within the 3 months prior to imaging; (4) pregnancy; or (5) a history of head trauma.

All participants provided written informed consent, and informed written consent was also obtained from the guardians of two participants aged 17 years.

#### Ethics Approval Statement

The protocol for study was approved by the Ethics Committee of the Medical Faculty of Fourth Military Medical University (registry no. KY20143344-1). The study adhered to the ethical principles for medical research as stated in the Declaration of Helsinki.

### Method details

#### Neuropsychological tests

All study participants completed in-depth neuropsychological assessments that evaluated a wide range of cognitive functions through collection of the following data: (1) visual memory data, including IVIM and DVIM as measures of working memory of figures and shapes; (2) verbal memory data, including IVBM and delayed verbal memory (DVBM) as measures of working memory of words; (3) simple reaction time data, including VSRT and auditory simple reaction time (ASRT) as indicators of psychomotor function in response to a simple stimulus; and (4) recognition reaction time data, including visual recognition reaction time (VCRT) and auditory recognition reaction time (ACRT) as measures of psychomotor function in response to target/distractive stimuli. The Chinese version of CNS Vital Signs program was used for all neuropsychological testing.^34^

#### MRI data acquisition

MRI examinations for the experimental group were conducted using a General Electric Discovery MR750 3.0-T (General Electric Co. Ltd., CT) scanner in Xijing Hospital of the Fourth Military Region. Standard T1-weighted, three-dimensional anatomical data were obtained using the Spoiled Gradient Recalled Echo sequence, with the following parameters: repetition time = 2530 ms, echo time = 3.5 ms, flip angle = 7°, resolution matrix = 256 × 256, number of slices = 192, slice thickness = 1.0 mm, and voxel size = 1.0 × 1.0 × 1.0 mm^3^. Functional MRI (fMRI) data were acquired with an echo planar imaging sequence covering the entire brain, with the following parameters: repetition time = 2000 ms; echo time = 30 ms; flip angle = 90°; field of view = 220 mm × 220 mm; acquisition matrix = 64 × 64; slice thickness = 4 mm; number of slices = 30, voxel size = 3.5 × 3.5 × 4.0 mm^3^, and number of volumes = 180.

MRI examinations for the control group were completed using a 3.0-T Siemens Trio MRI scanner (Siemens Medical, Erlangen, Germany) in the Southwest University Center for Brain Imaging. High-resolution T1-weighted anatomical images were acquired using a magnetization -prepared rapid gradient echo sequence, with the following parameters: repetition time = 1900 ms, echo time = 2.52 ms, inversion time = 900 ms, flip angle = 9°, resolution matrix = 256 × 256, number of slices = 176, slice thickness = 1.0 mm, and voxel size = 1.0 × 1.0 × 1.0 mm^3^. fMRI data were obtained using the echo planar imaging sequence, with the following parameters: repetition time = 2000 ms; echo time = 30 ms; flip angle = 90°, field of view = 220 × 220 mm^2^, slice thickness = 3 mm; slice gap = 1 mm, number of slices = 30, and voxel size = 3.4 × 3.4 × 3 mm^3^.

#### Resting-state fMRI data preprocessing

The Resting-State fMRI Data Analysis Toolkit Plus (RESTplus, http://www.restfmri.net/forum) was used for rs-fMRI data preprocessing. The following procedures were applied: conversion from the Digital Imaging and Communications in Medicine (DICOM) format to the Neuroimaging Informatics Technology Initiative (NIfTI) format; removal of the first 10 points; slice timing; realignment for head motion correction (participants with displacement exceeding 2 mm in 3D space or a rotation angle exceeding 2° were excluded); spatial normalization with resampling of 3 × 3 × 3 mm^3^ volumes to the Montreal Neurological Institute (MNI) space; spatial smoothing with a 6-mm full-width at half maximum (FWHM) Gaussian kernel; removal of linear detrending; regression for nuisance covariates; and band-pass filtering at 0.01–0.08 Hz.

#### Local activity metric determination

ALFF and fALFF maps were calculated without a band-pass filtering procedure. Each time series was first converted into a frequency domain power spectrum using the fast Fourier transform method. For calculation of the square root of the power spectrum, voxels within the range of 0.01–0.08 Hz were averaged. The resulting average square root of the power spectrum was recorded as the ALFF. Next the average ALFF value at the whole-brain voxel level was subtracted from the voxel-level ALFF map for each participant and then divided by the standard deviation. Using this value, the ratio of the root-mean-square of the power spectrum in the low-frequency range (0.01–0.08 Hz) to that for the whole frequency was calculated to determine the fALFF. Z transformation was then applied to obtain standardized ALFF and fALFF maps. Additionally, ReHo was calculated as a metric of regional functional synchronization. After realignment, slice timing, normalization, and regression, individual ReHo maps were generated, transformed into Z-scores, and then spatially smoothed with a 6-mm FWHM Gaussian kernel.

#### Static FC analysis

The Graph Theoretical Network Analysis (GRETNA) toolbox was used to construct sFC networks.^35^ We applied a 116-region of interest (ROI) atlas with a radius of 3 mm to calculate sFC in this study. For this analysis, the entire cerebral and cerebellar gray matter area was separated into 116 anatomically defined regions based on the Automated Anatomical Labeling (AAL) atlas.^39^ Next, upon extraction of the mean time series for each of the 116 regions, Pearson’s correlation coefficients were computed to generate a 116 × 116 FC matrix for each participant. Fisher’s Z transformation was performed to convert the data into z-values, which closely followed a normal distribution.

The rs-fMRI signal for each node was calculated by averaging the preprocessed signals of all voxels within a given ROI. The Fisher’s Z-transformed Pearson correlation coefficients between the rs-fMRI time series defined the edges between all possible node pairs.

#### Dynamic FC analysis

The sliding-time window approach was applied to generate dFC networks, using the AAL-116 as the ROI template within the DynamicBC toolbox, which captures correlations between time series over specific time ranges.^40^ The resulting 61-window FC matrices represent the dynamic changes in FC throughout the entire fMRI scanning process. For this study, we used a window length of 50 repetition times (100 s) to calculate the temporal variability of the dFC network (DFCN). For each dataset, the dynamic time window sequence consisted of 61 repetition times (61 s) with a window offset of 1 repetition time (1 s).

After dFC calculation, we applied *k*-means clustering to identify a set of dFC states that partitioned the data into separate clusters with the goal of maximizing the correlation within a cluster to the cluster centroid. Through this clustering analysis, we used the L1 distance (Manhattan distance) to estimate the similarity between two dFC matrices, because the L1 distance provides an effective similarity measure for high-dimensional data. Before the clustering analysis, we used the elbow criterion, defined as the ratio of within-cluster to between-cluster distances to determine the optimal number of clusters. This method produced a *k* value of 2 within a search window of *k* from 2 to 20.

We further compared dFC states among three timepoints based on calculated values for the NT (defined as number of transitions from a specific state to another state), MDT (duration of an individual dFC state), FT (total number of windows belonging to a state), SDFCN (intensity response of each based on the FC median matrix corresponding to each state), and VDFCN (variability response of each state based on the variance matrix corresponding to each state).

### Quantification and statistical analysis

#### Statistical analysis

SPSS 29.0 software (SPSS, Inc.) was used to analyze demographic, neuropsychological, and physiological data in this study. Subjective ratings of memory and reaction time were compared using one-way repeated-measures ANOVA across the three longitudinal time points (Time 1, Time 2, and Time 3), and *post-hoc* Bonferroni’s test was applied with a within-subject factor of condition in the experimental group. Partial eta-squared (η_p_^2^) values were calculated to reflect effect size.

For imaging metrics (ALFF, fALFF, ReHo, sFC and dFC), one-way repeated-measures ANOVA was used to evaluate longitudinal changes across the three time points in both the experimental and control groups. To control for high-dimensional multiple comparisons and Type I groups, FDR correction was applied at a threshold of *Q* = 0.05. For confirmation of differences, post-hoc pairwise comparisons between time points were further conducted using paired *t-*tests, with FDR correction also implemented at *p* < 0.05 to ensure rigor. For brain regions and nodes showing significant main effects in the ANOVA, we extracted the mean time series within each participant, and longitudinal temporal changes were evaluated using MATLAB (R2023a). Partial eta-squared (η_p_^2^) values were calculated to reflect effect size.

For any measure indicating a cognition-related alteration, Pearson correlation analysis was employed to detect the correlation between changes in a brain functional index and changes in a neuropsychological feature. A correlation was considered significant if *p* < 0.05.
