# Supplementary material for: Dynamic functional connectivity variability may explain hypoxia-induced cognitive impairment at high-altitude
Source: iScience. 2026 Aug 3;29(8):117015. doi: 10.1016/j.isci.2026.117015 (PMC13470281; doi:10.1016/j.isci.2026.117015)

iScience, Volume *29*

Supplemental information

Dynamic functional connectivity variability may explain hypoxia-induced cognitive impairment at high-altitude

Siyao Zeng, Sijia Guo, Yang Zhou, Jiebing He, Yang Ou, Tingting Yang, Jia Wang, Wensheng Zhai, Baojuan Li, Jie Liu, Wenjing Luo, and Xiaoming Chen

Supplementary Table 1. Descriptive Values and Statistical Analysis of Altered Physiological parameters before and after High-altitude Exposure (*n*=49)

Physiological parameters	Time 1 (mean±SD)	Time 2 (mean±SD)	Time 3 (mean±SD)	*F*	*p*	ηp^2	Post-hoc comparison
RBC, 10^9/L	4.752±0.492	5.660±0.495	5.549±0.509	164.637	<0.001	0.774	Time 1<Time 2 (*p*<0.001)<br>Time 1<Time 3 (*p*<0.001)
HBG, g/L	148.542±14.226	170.714±15.022	171.100±15.624	97.552	<0.001	0.670	Time 1<Time 2 (*p*<0.001)<br>Time 1<Time 3 (*p*<0.001)
HR, b/m	82.939±8.906	91.061±11.771	90.470±11.017	19.622	<0.001	0.290	Time 1<Time 2 (*p*<0.001)<br>Time 1<Time 3 (*p*<0.001)
SBP, mmHg	106.531±14.011	115.245±11.110	110.796±11.934	10.890	<0.001	0.185	Time 1<Time 2 (*p*<0.001)<br>Time 2>Time 3 (*p*=0.038)
DBP, mmHg	65.898±8.471	69.960±10.210	74.714±10.120	17.043	<0.001	0.262	Time 1<Time 2 (*p*=0.022)<br>Time 1<Time 3 (*p*<0.001)<br>Time 2<Time 3 (*p*=0.007)
LVEF, %	55.939±6.084	68.083±3.558	67.286±2.300	119.741	<0.001	0.714	Time 1<Time 2 (*p*<0.001)<br>Time 1<Time 3 (*p*<0.001)
LVFS, %	30.245±3.827	37.875±2.728	37.347±1.653	110.91	<0.001	0.698	Time 1<Time 2 (*p*<0.001)<br>Time 1<Time 3 (*p*<0.001)
SpO2, %	97.582±0.612	87.878±3.094	88.751±2.879	235.679	<0.001	0.831	Time 1>Time 2 (*p*<0.001)<br>Time 1>Time 3 (*p*<0.001)
RR, /min	20.408±2.541	22.306±4.346	22.020±3.778	8.891	<0.001	0.156	Time 1<Time 2 (*p*=0.007)<br>Time 1<Time 3 (*p*=0.015)
VC, L	4.085±0.876	3.787±0.712	3.549±0.694	73.409	<0.001	0.605	Time 1>Time 2 (*p*<0.001)<br>Time 1>Time 3 (*p*<0.001)<br>Time 2>Time 3 (*p*<0.001)
FVC, L	4.078±0.871	3.710±0.697	3.540±0.702	74.907	<0.001	0.609	Time 1>Time 2 (*p*<0.001)<br>Time 1>Time 3 (*p*<0.001)<br>Time 2>Time 3 (*p*<0.001)
FEV1, L	3.612±0.714	3.395±0.665	3.262±0.659	26.477	<0.001	0.356	Time 1>Time 2 (*p*=0.017)<br>Time 1>Time 3 (*p*<0.001)<br>Time 2>Time 3 (*p*<0.001)

Note: DBP, diastolic blood pressure; FEV1, forced expiratory volume in one second; FVC, forced vital capacity; HR, heart rate; HBG, hemoglobin concentration; LVEF, left ventricular ejection fractions; LVFS, left ventricular fractional shortening; SpO2, peripheral oxygen saturation; SBP, systolic blood pressure; RBC, red blood cell count; RR, respiratory rate; VC, vital capacity.

Supplementary Table 2. Brain Regions with ALFF Differences before and after High-altitude Exposure (*n*=49)

One-way Repeated Measures ANOVA [a]				Paired *t*-test [b] (Time 1 *vs.* Time 2)			Paired *t*-test [b] (Time 2 *vs.* Time 3)		
Brain regions	*F*	*p*	ηp²	Brain regions	*t*	*p*	Brain regions	*t*	*p*
PreCG.R	8.872	<0.001	0.110	PreCG.R	-3.343	0.002	PreCG.R	6.731	<0.001
SMA.L	9.450	<0.001	0.116	SMA.L	-2.453	0.018	SMA.L	5.958	<0.001
SMA.R	8.410	<0.001	0.105	HIP.L	4.932	<0.001	SMA.R	6.047	<0.001
HIP.L	14.674	<0.001	0.169	HIP.R	4.960	<0.001	HIP.L	-6.118	<0.001
HIP.R	12.672	<0.001	0.150	CAU.L	6.020	<0.001	HIP.R	-5.534	<0.001
CAU.L	11.650	<0.001	0.139	CAU.R	4.928	<0.001	CAU.L	-4.415	<0.001
CAU.R	11.596	<0.001	0.139	THA.R	4.064	<0.001	CAU.R	-4.449	<0.001
THA.R	8.206	<0.001	0.102	STG.R	-5.127	<0.001	STG.R	6.051	<0.001
STG.R	11.239	<0.001	0.135	MTG.R	-3.410	0.001	MTG.R	4.866	<0.001
MTG.R	7.633	0.001	0.096	CRBL3.R	4.996	<0.001	CRBL3.R	-3.134	0.003
CRBL3.R	7.306	0.001	0.092	CRBL9.L	5.582	<0.001	CRBL9.L	-4.962	<0.001
CRBL9.L	9.107	<0.001	0.112	CRBL9.R	7.252	<0.001	CRBL9.R	-5.658	<0.001
CRBL9.R	15.687	<0.001	0.179	Vermis9	5.085	<0.001	Vermis9	-4.487	<0.001
Vermis9	8.991	<0.001	0.111	Vermis10	7.069	<0.001	Vermis10	-5.587	<0.001
Vermis10	15.562	<0.001	0.178	-	-	-	-	-	-

Note: [a] uncorrect, p<0.001; [b] FDR corrected, p<0.05. Abbreviations: ALFF, amplitude of low frequency fluctuations; CAU.L, left caudate nucleus; CAU.R, right caudate nucleus; CRBL3.R, right cerebelum_3; CRBL9.L, left cerebelum_9; CRBL9.R, right cerebelum_9; HIP.L, left hippocampus; HIP.R, right hippocampus; MTG.R, right middle temporal gyrus; PreCG.R, right precental gyrus; SMA.L, left supplementary motor area; SMA.R, right supplementary motor area; STG.R, right superior temporal gyrus; THA.R, right thalamus.

Supplementary Table 3. Brain Regions with fALFF Differences before and after High-altitude Exposure (*n*=49)

One-way Repeated Measures ANVOA [a]				Paired *t*-test [b] (Time 1 *vs.* Time 2)			Paired *t*-test [b] (Time 2 *vs.* Time 3)		
Brain regions	*F*	*p*	ηp^2	Brain regions	*t*	*p*	Brain regions	*t*	*p*
PreCG.R	7.571	0.001	0.095	PreCG.R	-3.933	<0.001	PreCG.R	4.094	<0.001
ROL.R	7.430	0.001	0.094	ROL.R	-4.711	<0.001	ROL.R	3.757	<0.001
SMA.L	11.424	<0.001	0.137	SMA.L	-4.450	<0.001	SMA.L	4.817	<0.001
DCG.L	7.565	0.001	0.095	DCG.L	-4.041	<0.001	DCG.L	2.848	0.006
HIP.L	12.051	<0.001	0.143	HIP.L	4.815	<0.001	HIP.L	-4.456	<0.001
HIP.R	15.806	<0.001	0.180	HIP.R	5.321	<0.001	HIP.R	-5.017	<0.001
PHG.L	10.437	<0.001	0.127	PHG.L	4.687	<0.001	PHG.L	-4.148	<0.001
PHG.R	9.585	<0.001	0.117	PHG.R	5.149	<0.001	PHG.R	-3.446	0.001
PCL.R	8.678	<0.001	0.108	HES.R	-6.228	<0.001	PCL.R	5.391	<0.001
HES.R	15.137	<0.001	0.174	TPOmid.R	4.621	<0.001	HES.R	5.074	<0.001
TPOmid.R	8.041	<0.001	0.100	CRBL6.R	-3.507	0.001	CRBL6.R	3.363	0.002
CRBL6.R	7.436	0.001	0.094	CRBL9.L	4.647	<0.001	CRBL9.L	-4.231	<0.001
CRBL9.L	8.916	<0.001	0.110	CRBL9.R	4.474	<0.001	CRBL9.R	-4.076	<0.001
CRBL9.R	8.518	<0.001	0.106	-	-	-	-	-	-

Note: [a] uncorrect, $p<0.001$; [b] FDR corrected, $p<0.05$. Abbreviations: fALFF, fractional amplitude of low frequency fluctuations; CRBL6.R, right cerebelum_6; CRBL9.L, left cerebelum_9; CRBL9.R, right cerebelum_9; DCG.L, left median cingulate and paracingulate gyri; HIP.L, left hippocampus; HIP.R, right hippocampus; HES.R, right heschl gyrus; PCL.R, right paracentral lobule; PHG.L, left parahippocampal gyrus; PHG.R, right parahippocampal gyrus; PreCG.R, right precental gyrus; ROL.R, right rolandic operculum; SMA.L, left supplementary motor area; TPOmid.R, right temporal pole: middle temporal gyrus.

Supplementary Table 4. Brain Regions with ReHo Differences before and after High-altitude Exposure (*n*=49)

One-way Repeated Measures ANOVA [a]				Paired *t*-test [b] (Time 1 *vs.* Time 2)			Paired *t*-test [b] (Time 2 *vs.* Time 3)			Paired *t*-test [b] (Time 1 *vs.* Time 3)		
Brain regions	*F*	*p*	ηp^2	Brain regions	*t*	*p*	Brain regions	*t*	*p*	Brain regions	*t*	*p*
ORBmid.R	7.507	0.001	0.094	ORBmid.R	4.195	<0.001	ORBmid.R	-3.730	0.001	INS.L	-3.944	<0.001
SMA.R	10.675	<0.001	0.129	SMA.R	-3.816	<0.001	SMA.R	5.173	<0.001	PUT.L	-3.901	<0.001
INS.L	9.746	<0.001	0.119	INS.L	-4.942	<0.001	DCG.L	5.641	<0.001	PUT.R	-2.980	0.005
DCG.L	23.118	<0.001	0.243	DCG.L	-6.407	<0.001	DCG.R	4.291	<0.001	PAL.L	-3.477	0.001
DCG.R	10.095	<0.001	0.123	DCG.R	-4.343	<0.001	PCL.L	4.076	<0.001	CRBL45.L	-2.491	0.016
PCL.L	7.547	0.001	0.095	PUT.L	-5.414	<0.001	HES.L	4.833	<0.001	CRBL6.L	-2.367	0.022
PUT.L	12.525	<0.001	0.148	PUT.R	-4.420	<0.001	HES.R	5.295	<0.001	Vermis6	-2.905	0.006
PUT.R	8.406	<0.001	0.105	PAL.L	-5.013	<0.001	CRBL45.L	3.027	0.004	Vermis7	-2.746	0.008
PAL.L	9.405	<0.001	0.116	PAL.R	-4.607	<0.001	CRBL45.R	4.168	<0.001	-	-	-
PAL.R	7.440	0.001	0.094	HES.L	-5.300	<0.001	CRBL6.L	2.645	0.011	-	-	-
HES.L	13.459	<0.001	0.157	HES.R	-6.000	<0.001	CRBL6.R	4.566	<0.001	-	-	-
HES.R	15.339	<0.001	0.176	CRBL45.L	-5.318	<0.001	-	-	-	-	-	-
CRBL45.L	10.655	<0.001	0.129	CRBL45.R	-5.523	<0.001	-	-	-	-	-	-
CRBL45.R	10.326	<0.001	0.125	CRBL6.L	-4.509	<0.001	-	-	-	-	-	-
CRBL6.L	8.534	<0.001	0.106	CRBL6.R	-5.158	<0.001	-	-	-	-	-	-
CRBL6.R	11.762	<0.001	0.140	Vermis6	-4.876	<0.001	-	-	-	-	-	-
Vermis6	11.430	<0.001	0.137	Vermis7	-3.839	<0.001	-	-	-	-	-	-
Vermis7	7.329	0.001	0.092	-	-	-	-	-	-	-	-	-

Note: [a] uncorrect, $p<0.001$; [b] FDR corrected, $p<0.05$. Abbreviations: ReHo, regional homogeneity; CRBL6.L, left cerebelum_6; CRBL6.R, right cerebelum_6; CRBL45.L, left cerebelum_4_5; CRBL45.R, right cerebelum_4_5; DCG.L, left median cingulate and paracingulate gyri; DCG.R, right median cingulate and paracingulate gyri; HES.L, left heschl gyrus; HES.R, right heschl gyrus; INS.L, left insula; ORBmid.R, right middle frontal gyrus, orbital part; PCL.L, left paracentral lobule; PUT.L, left lenticular nucleus, putamen; PUT.R, right lenticular nucleus, putamen; PAL.L, left lenticular nucleus, pallidum; PAL.R, right lenticular nucleus, pallidum; SMA.R, right supplementary motor area.

Supplementary Table 5. One-way Repeated Measures ANOVA of Alterations in sFC before and after High-altitude Exposure (n=49)

Brain regions	Z1	Z2	Z3	F	p	ηp^2
ORBsupmed.R–REC.R	0.762	0.633	0.698	8.187	<0.001	0.102
PreCG.L–ACG.L	0.315	0.143	0.317	7.980	0.001	0.100
SMA.L–ACG.L	0.514	0.326	0.473	11.366	<0.001	0.136
REC.L–ACG.L	0.574	0.419	0.507	7.991	0.001	0.100
REC.R–ACG.L	0.554	0.386	0.508	9.103	<0.001	0.112
OLF.L–PHG.L	0.482	0.325	0.450	7.920	0.001	0.099
ACG.L–PHG.L	0.493	0.321	0.420	10.234	<0.001	0.124
ACG.L–PHG.R	0.461	0.306	0.441	9.490	<0.001	0.116
HIP.L–AMYG.L	0.658	0.550	0.634	7.832	0.001	0.098
ACG.L–AMYG.R	0.396	0.240	0.379	8.154	<0.001	0.102
ACG.L–PCL.L	0.362	0.197	0.372	11.822	<0.001	0.141
ACG.L–PCL.R	0.339	0.202	0.342	7.846	0.001	0.098
HIP.L–PUT.L	0.447	0.275	0.382	8.802	<0.001	0.109
HIP.R–PUT.L	0.436	0.265	0.389	8.730	<0.001	0.108
PHG.L–PUT.L	0.413	0.233	0.342	8.271	<0.001	0.103
PCL.L–PUT.L	0.426	0.275	0.339	9.578	<0.001	0.117
PreCG.R–PUT.R	0.502	0.347	0.463	8.635	<0.001	0.107
SMA.R–PUT.R	0.587	0.462	0.557	8.880	<0.001	0.110
INS.L–PUT.R	0.619	0.492	0.577	7.903	0.001	0.099
INS.R–PUT.R	0.660	0.526	0.616	10.058	<0.001	0.123
HIP.L–PUT.R	0.400	0.226	0.317	8.291	<0.001	0.103
PHG.L–PUT.R	0.403	0.200	0.298	9.269	<0.001	0.114
PCL.L–PUT.R	0.400	0.235	0.346	10.103	<0.001	0.123
SMA.R–PAL.R	0.570	0.395	0.534	15.484	<0.001	0.177
ACG.L–HES.L	0.393	0.174	0.364	11.409	<0.001	0.137
ACG.R–HES.L	0.378	0.163	0.353	11.533	<0.001	0.138
PUT.L–HES.L	0.495	0.331	0.377	9.713	<0.001	0.119
ROL.R–HES.R	0.741	0.611	0.714	9.721	<0.001	0.119
INS.L–HES.R	0.597	0.434	0.562	8.977	<0.001	0.111
INS.R–HES.R	0.676	0.513	0.644	10.652	<0.001	0.129
ACG.L–HES.R	0.422	0.229	0.368	8.000	0.001	0.100
PUT.L–HES.R	0.505	0.291	0.376	14.593	<0.001	0.169
PUT.R–HES.R	0.553	0.336	0.435	13.979	<0.001	0.163
PAL.L–HES.R	0.438	0.220	0.311	11.805	<0.001	0.141
PAL.R–HES.R	0.440	0.235	0.333	10.818	<0.001	0.131
THA.R–HES.R	0.370	0.158	0.287	8.340	<0.001	0.104
ACG.L–STG.L	0.372	0.146	0.327	9.982	<0.001	0.122
ACG.R–STG.L	0.352	0.144	0.324	8.484	<0.001	0.105
HES.R–STG.R	0.721	0.606	0.732	10.839	<0.001	0.131
ACG.L–TPOsup.L	0.517	0.280	0.437	15.110	<0.001	0.173
ACG.R–TPOsup.L	0.473	0.267	0.403	10.132	<0.001	0.123
PUT.L–TPOsup.L	0.423	0.234	0.344	11.383	<0.001	0.137
ACG.L–TPOsup.R	0.434	0.203	0.354	10.847	<0.001	0.131
ACG.R–TPOsup.R	0.434	0.202	0.356	10.767	<0.001	0.130

HIP.L–TPOsup.R	0.419	0.251	0.401	8.239	<0.001	0.103
PUT.L–TPOsup.R	0.389	0.180	0.326	15.754	<0.001	0.180
PUT.R–TPOsup.R	0.433	0.216	0.380	16.293	<0.001	0.185
PAL.L–TPOsup.R	0.320	0.127	0.268	9.096	<0.001	0.112
PAL.R–TPOsup.R	0.360	0.160	0.303	11.284	<0.001	0.135
HES.L–TPOsup.R	0.552	0.413	0.580	9.824	<0.001	0.120
HES.R–TPOsup.R	0.515	0.364	0.549	9.120	<0.001	0.112
ACG.L–TPOmid.R	0.406	0.244	0.328	7.942	0.001	0.099
ACG.L–ITG.R	0.394	0.235	0.276	7.895	0.001	0.099
PUT.L–CRBL3.R	0.077	-0.101	-0.010	9.008	<0.001	0.111
PUT.R–CRBL3.R	0.100	-0.095	0.002	10.418	<0.001	0.126
PUT.L–CRBL45.L	0.342	0.174	0.186	8.931	<0.001	0.110
PUT.R–CRBL45.L	0.344	0.181	0.189	8.653	<0.001	0.107
CUN.L–CRBL45.R	0.602	0.445	0.576	8.651	<0.001	0.107
LING.L–CRBL45.R	0.687	0.533	0.649	9.644	<0.001	0.118
LING.R–CRBL45.R	0.712	0.582	0.688	8.013	0.001	0.100
PUT.L–CRBL45.R	0.332	0.172	0.188	8.950	<0.001	0.111
PUT.R–CRBL45.R	0.328	0.171	0.188	8.195	<0.001	0.102
PUT.L–CRBL6.R	0.406	0.257	0.247	8.240	<0.001	0.103
HES.R–CRBL10.R	0.287	0.136	0.108	8.784	<0.001	0.109
ACG.L–Vermis3	0.235	0.084	0.206	8.501	<0.001	0.106
ACG.R–Vermis3	0.215	0.051	0.183	9.435	<0.001	0.116
DCG.R–Vermis3	0.212	0.063	0.230	8.596	<0.001	0.107
PUT.L–Vermis3	0.205	0.002	0.161	10.553	<0.001	0.128
PUT.R–Vermis3	0.234	0.010	0.154	11.698	<0.001	0.140
ORBsupmed.R–Vermis6	0.308	0.154	0.177	8.189	<0.001	0.102
PUT.L–Vermis6	0.335	0.183	0.249	7.908	0.001	0.099
PUT.R–Vermis6	0.338	0.192	0.243	7.925	0.001	0.099
SMA.R–Vermis7	0.372	0.224	0.240	8.078	<0.001	0.101
PUT.R–Vermis7	0.347	0.184	0.203	9.531	<0.001	0.117
Vermis6–Vermis7	0.737	0.588	0.653	13.093	<0.001	0.154
SMA.L–Vermis8	0.350	0.252	0.171	8.603	<0.001	0.107
PUT.L–Vermis8	0.362	0.192	0.218	8.872	<0.001	0.110
PUT.R–Vermis8	0.360	0.164	0.198	11.048	<0.001	0.133
PAL.R–Vermis8	0.375	0.227	0.246	8.315	<0.001	0.104
CRBL10.R–Vermis8	0.394	0.235	0.411	7.866	0.001	0.098

Note: uncorrect, $p<0.001$. Z1, Z2 and Z3 indicated the mean values of brain sFC before and after high-altitude exposure (Z1: Time 1; Z2: Time 2; Z3: Time 3). Abbreviations: ACG.L, left anterior cingulate and paracingulate gyri; ACG.R, right anterior cingulate and paracingulate gyri; AMYG. L, left amygdala; AMYG.R, right amygdala; CRBL3.R, right cerebelum_3; CRBL6.R, right cerebelum_6; CRBL10.R, right cerebelum_10; CRBL45.L, left cerebelum_4_5; CRBL45.R, right cerebelum_4_5; CUN.L, left cuneus; DCG.R, right median cingulate and paracingulate gyri; HIP.L, left hippocampus; HIP.R, right hippocampus; HES.L, left heschl gyrus; HES.R, right heschl gyrus; INS.L, left insula; INS.R, right insula; ITG.R, right inferior temporal gyrus; LING.L, left lingual gyrus; LING.R, right lingual gyrus; OLF.L, left olfactory cortex; ORBsupmed.R, right superior frontal gyrus, medial orbital; PreCG.L, left precental gyrus; PreCG.R, right precental gyrus; PHG.L, left parahippocampal gyrus; PHG.R, right parahippocampal gyrus; PCL.L, left paracentral lobule; PCL.R, right paracentral lobule; PUT.L, left lenticular nucleus, putamen; PUT.R, right lenticular nucleus, putamen; PAL.L, left lenticular nucleus, pallidum; PAL.R, right lenticular nucleus, pallidum; ROL.R, right rolandic operculum; REC.L, left gyrus rectus; REC.R, right gyrus rectus; SMA.L, left supplementary motor area; SMA.R, right supplementary motor area; STG.L, left superior temporal gyrus; STG.R, right superior temporal gyrus; THA.R, right thalamus; TPOsup.L, left temporal

pole: superior temporal gyrus; TPOsup.R, right temporal pole: superior temporal gyrus; TPOmid.R, right temporal pole: middle temporal gyrus; sFC, state functional connectivity.

Supplementary Table 6. One-way Repeated Measures ANOVA of Alterations in sFC with Paired *t*-test Results between Time 1 and Time 2 (*n*=49)

Brain regions	*t*	*p*
ORBsupmed.R-REC.R	-4.334	<0.001
PreCG.L–ACG.L	-4.114	<0.001
SMA.L–ACG.L	-6.465	<0.001
REC.L–ACG.L	-4.829	<0.001
REC.R–ACG.L	-5.378	<0.001
OLF.L–PHG.L	-4.123	<0.001
ACG.L–PHG.L	-5.300	<0.001
ACG.L–PHG.R	-4.531	<0.001
HIP.L–AMYG.L	-4.899	<0.001
ACG.L–AMYG.R	-3.775	<0.001
ACG.L–PCL.L	-4.410	<0.001
ACG.L–PCL.R	-3.945	<0.001
HIP.L–PUT.L	-4.357	<0.001
HIP.R–PUT.L	-4.044	<0.001
PHG.L–PUT.L	-4.028	<0.001
PCL.L–PUT.L	-4.882	<0.001
PreCG.R–PUT.R	-6.193	<0.001
SMA.R–PUT.R	-4.664	<0.001
INS.L–PUT.R	-4.055	<0.001
INS.R–PUT.R	-4.700	<0.001
HIP.L–PUT.R	-4.714	<0.001
PHG.L–PUT.R	-4.755	<0.001
PCL.L–PUT.R	-4.847	<0.001
SMA.R–PAL.R	-6.033	<0.001
ACG.L–HES.L	-5.330	<0.001
ACG.R–HES.L	-5.593	<0.001
PUT.L–HES.L	-4.416	<0.001
ROL.R–HES.R	-4.765	<0.001
INS.L–HES.R	-4.439	<0.001
INS.R–HES.R	-5.483	<0.001
ACG.L–HES.R	-5.093	<0.001
PUT.L–HES.R	-5.515	<0.001
PUT.R–HES.R	-5.474	<0.001
PAL.L–HES.R	-4.559	<0.001
PAL.R–HES.R	-4.702	<0.001
THA.R–HES.R	-4.396	<0.001
ACG.L–STG.L	-5.248	<0.001
ACG.R–STG.L	-4.898	<0.001
HES.R–STG.R	-3.978	<0.001
ACG.L–TPOsup..L	-6.557	<0.001
ACG.R–TPOsup.L	-5.549	<0.001
PUT.L–TPOsup.L	-4.732	<0.001
ACG.L–TPOsup.R	-5.442	<0.001
ACG.R–TPOsup.R	-5.628	<0.001

HIP.L–TPOsup.R	-3.697	0.001
PUT.L–TPOsup.R	-5.473	<0.001
PUT.R–TPOsup.R	-5.021	<0.001
PAL.L–TPOsup.R	-4.137	<0.001
PAL.R–TPOsup.R	-4.636	<0.001
HES.L–TPOsup.R	-3.688	0.001
HES.R–TPOsup.R	-3.158	0.003
ACG.L–TPOmid.R	-4.461	<0.001
ACG.L–ITG.R	-4.376	<0.001
PUT.L–CRBL3.R	-4.530	<0.001
PUT.R–CRBL3.R	-4.912	<0.001
PUT.L–CRBL45.L	-4.441	<0.001
PUT.R–CRBL45.L	-4.435	<0.001
CUN.L–CRBL45.R	-4.461	<0.001
LING.L–CRBL45.R	-4.318	<0.001
LING.R–CRBL45.R	-4.142	<0.001
PUT.L–CRBL45.R	-4.766	<0.001
PUT.R–CRBL45.R	-4.637	<0.001
PUT.L–CRBL6.R	-3.585	0.001
HES.R–CRBL10.R	-3.238	0.002
ACG.L–Vermis3	-3.764	<0.001
ACG.R–Vermis3	-3.965	<0.001
DCG.R–Vermis3	-3.224	0.002
PUT.L–Vermis3	-5.118	<0.001
PUT.R–Vermis3	-5.278	<0.001
ORBsupmed.R–Vermis6	-4.611	<0.001
PUT.L–Vermis6	-4.337	<0.001
PUT.R–Vermis6	-4.505	<0.001
SMA.R–Vermis7	-3.745	<0.001
PUT.R–Vermis7	-5.215	<0.001
Vermis6–Vermis7	-5.206	<0.001
PUT.L–Vermis8	-4.702	<0.001
PUT.R–Vermis8	-5.647	<0.001
PAL.R–Vermis8	-4.351	<0.001
CRBL10.R–Vermis8	-3.780	<0.001

Note: FDR corrected, $p<0.05$. Abbreviations: sFC, state functional connectivity; ACG.L, left anterior cingulate and paracingulate gyri; ACG.R, right anterior cingulate and paracingulate gyri; AMYG.L, left amygdala; AMYG.R, right amygdala; CRBL3.R, right cerebelum_3; CRBL6.R, right cerebelum_6; CRBL10.R, right cerebelum_10; CRBL45.L, left cerebelum_4_5; CRBL45.R, right cerebelum_4_5; CUN.L, left cuneus; DCG.R, right median cingulate and paracingulate gyri; HIP.L, left hippocampus; HIP.R, right hippocampus; HES.L, left heschl gyrus; HES.R, right heschl gyrus; INS.L, left insula; INS.R, right insula; ITG.R, right inferior temporal gyrus; LING.L, left lingual gyrus; LING.R, right lingual gyrus; OLF.L, left olfactory cortex; ORBsupmed.R, right superior frontal gyrus, medial orbital; PreCG.L, left precental gyrus; PreCG.R, right precental gyrus; PHG.L, left parahippocampal gyrus; PHG.R, right parahippocampal gyrus; PCL.L, left paracentral lobule; PCL.R, right paracentral lobule; PUT.L, left lenticular nucleus, putamen; PUT.R, right lenticular nucleus, putamen; PAL.L, left lenticular nucleus, pallidum; PAL.R, right lenticular nucleus, pallidum; ROL.R, right rolandic operculum; REC.L, left gyrus rectus; REC.R, right gyrus rectus; SMA.L, left supplementary motor area; SMA.R, right supplementary motor area; STG.L, left superior temporal gyrus; STG.R, right superior temporal gyrus; THA.R, right thalamus; TPOsup.L, left temporal pole: superior temporal gyrus; TPOsup.R, right temporal pole: superior temporal gyrus; TPOmid.R, right temporal pole: middle temporal gyrus.

Supplementary Table 7. One-way Repeated Measures ANOVA of Alterations in sFC with Paired *t*-test Results between Time 2 and Time 3 (*n*=49)

Brain regions	*t*	*p*
PreCG.L–ACG.L	3.805	<0.001
SMA.L–ACG.L	3.723	0.001
REC.R–ACG.L	3.142	0.003
OLF.L–PHG.L	3.223	0.002
ACG.L–PHG.R	3.349	0.002
HIP.L–AMYG.L	3.557	0.001
ACG.L–AMYG.R	3.055	0.004
ACG.L–PCL.L	4.779	<0.001
ACG.L–PCL.R	3.776	<0.001
INS.R-PUT.R	3.080	0.003
PCL.L–PUT.R	3.116	0.003
SMA.R–PAL.R	4.217	<0.001
ACG.L–HES.L	4.014	<0.001
ACG.R–HES.L	4.049	<0.001
ROL.R–HES.R	4.031	<0.001
INS.L–HES.R	3.846	<0.001
INS.R–HES.R	4.152	<0.001
ACG.L–HES.R	3.088	0.003
ACG.L–STG.L	3.070	0.004
ACG.R–STG.L	3.127	0.003
HES.R–STG.R	4.798	<0.001
ACG.L–TPOsup.L	3.517	0.001
ACG.R–TPOsup.R	3.040	0.004
HIP.L–TPOsup.R	3.633	0.001
PUT.L–TPOsup.R	3.457	0.001
PUT.R–TPOsup.R	3.674	0.001
PAL.R–TPOsup.R	3.160	0.003
HES.L–TPOsup.R	4.690	<0.001
HES.R–TPOsup.R	4.233	<0.001
CUN.L–CRBL45.R	3.047	0.004
LING.L–CRBL45.R	3.202	0.002
LING.R–CRBL45.R	3.173	0.003
ACG.R–Vermis3	3.093	0.003
DCG.R–Vermis3	4.241	<0.001
PUT.L–Vermis3	3.139	0.003
CRBL10.R–Vermis8	3.938	<0.001

Note: FDR corrected, $p<0.05$. Abbreviations: sFC, state functional connectivity; ACG.L, left anterior cingulate and paracingulate gyri; ACG.R, right anterior cingulate and paracingulate gyri; AMYG.L, left amygdala; AMYG.R, right amygdala; CRBL10.R, right cerebelum_10; CRBL45.R, right cerebelum_4_5; CUN.L, left cuneus; DCG.R, right median cingulate and paracingulate gyri; HIP.L, left hippocampus; HES.L, left heschl gyrus; HES.R, right heschl gyrus; INS.L, left insula; INS.R, right insula; LING.L, left lingual gyrus; LING.R, right lingual gyrus; OLF.L, left olfactory cortex; PreCG.L, left precental gyrus; PHG.L, left parahippocampal gyrus; PHG.R, right parahippocampal gyrus; PAL.R, right lenticular nucleus, pallidum; PCL.L, left paracentral lobule; PCL.R, right paracentral lobule; PUT.L, left lenticular nucleus, putamen; PUT.R, right lenticular nucleus, putamen; ROL.R, right rolandic operculum; REC.R, right gyrus rectus; SMA.L, left supplementary motor area; SMA.R, right supplementary motor area; STG.L, left superior temporal gyrus; STG.R, right superior temporal gyrus; TPOsup.L, left temporal pole: superior temporal gyrus; TPOsup.R, right temporal pole: superior temporal gyrus.

Supplementary Table 8. One-way repeated measures ANOVA of Alterations in sFC with Paired *t*-test Results between Time 1 and Time 3 (*n*=49)

Brain regions	*t*	*p*
PUT.L–HES.R	-3.465	0.001
PUT.R–HES.R	-3.612	0.001
PUT.L–CRBL45.L	-3.710	0.001
PUT.R–CRBL45.L	-3.717	0.001
PUT.L–CRBL45.R	-3.946	<0.001
PUT.R–CRBL45.R	-3.910	<0.001
PUT.L–CRBL6.R	-3.549	0.001
HES.R–CRBL10.R	-4.703	<0.001
ORBsupmed.R–Vermis6	-3.593	0.001
SMA.R–Vermis7	-3.725	0.001
SMA.L–Vermis8	-4.935	<0.001
PUT.R–Vermis8	-3.440	0.001

Note: FDR corrected, p<0.05. Abbreviations: sFC, state functional connectivity; CRBL6.R, right cerebelum_6; CRBL10.R, right cerebelum_10; CRBL45.L, left cerebelum_4_5; CRBL45.R, right cerebelum_4_5; HES.R, right heschl gyrus; ORBsupmed.R, right superior frontal gyrus, medial orbital; PUT.L, left lenticular nucleus, putamen; PUT.R, right lenticular nucleus, putamen; SMA.L, left supplementary motor area; SMA.R, right supplementary motor area.

Supplementary Table 9. One-way Repeated Measures ANOVA of Alterations in sFC in the Control Group (*n*=49)

Brain regions	Z1	Z2	Z3	*F*	*p*	ηp^2
SFGdor.L–CAL.L	0.561	0.444	0.541	8.253	<0.001	0.103
AMYG.L–CAL.L	0.354	0.243	0.342	7.475	0.001	0.094
SFGdor.L–CAL.R	0.506	0.380	0.485	8.703	<0.001	0.108
SFGmed.L–CAL.R	0.478	0.343	0.445	9.078	<0.001	0.112
ACG.L–CAL.R	0.392	0.243	0.363	7.712	0.001	0.097
AMYG.L–CAL.R	0.335	0.229	0.344	8.227	<0.001	0.103
SFGdor.L–CUN.R	0.526	0.414	0.478	7.345	0.001	0.093
AMYG.L–LING.L	0.322	0.215	0.299	7.489	0.001	0.094
AMYG.R–LING.L	0.319	0.229	0.301	7.345	0.001	0.093
AMYG.L–LING.R	0.353	0.260	0.389	7.291	0.001	0.092
PHG.L–Cere4_5.L	0.621	0.530	0.621	7.408	0.001	0.093
INS.L–Cere9.R	0.154	0.140	0.269	7.339	0.001	0.093
LING.L–Verm3	0.330	0.226	0.362	7.328	0.001	0.092

Note: uncorrected, $p<0.001$. Z1, Z2 and Z3 indicated the mean values of brain functional connectivity at three times (Z1: time 1; Z2: time 2; Z3: time 3). Abbreviations: sFC, state functional connectivity; AMYG.L, left amygdala; AMYG.R, right amygdala; ACG.L, left anterior cingulate and paracingulate gyri; CAL.L, left calcarine fissure and surrounding cortex; CAL.R, right calcarine fissure and surrounding cortex; CUN.R, right cuneus; Cere9.R, right cerebelum_9_; Cere4_5.L, left cerebelum_4_5_; INS.L, left insula; LING.L, left lingual gyrus; LING.R, right lingual gyrus; SFGdor.L, left superior frontal gyrus, dorsolateral; SFGmed.L, left superior frontal gyrus, medial.

Supplementary Table 10. One-way Repeated Measures ANOVA of Alterations in sFC with Paired *t*-test Results between Two Times in the Control Group (*n*=49)

Time 1 *vs.* Time 2			Time 2 *vs.* Time 3		
Brain regions	*t*	*p*	Brain regions	*t*	*p*
SFGdor.L–CAL.L	-3.889	<0.001	SFGdor.L–CAL.L	2.969	0.005
AMYG.L–CAL.L	-3.772	<0.001	AMYG.L–CAL.L	2.940	0.005
SFGdor.L–CAL.R	-3.989	<0.001	SFGdor.L–CAL.R	2.993	0.004
SFGmed.L–CAL.R	-4.415	<0.001	SFGmed.L–CAL.R	2.944	0.005
ACG.L–CAL.R	-4.161	<0.001	AMYG.L–CAL.R	3.239	0.002
AMYG.L–CAL.R	-3.374	0.001	AMYG.L–LING.L	3.267	0.002
SFGdor.L–CUN.R	-3.891	<0.001	AMYG.R–LING.L	3.270	0.002
AMYG.L–LING.L	-2.904	0.006	AMYG.L–LING.R	3.113	0.003
AMYG.R–LING.L	-3.372	0.001	PHG.L–Cere4_5.L	3.112	0.003
PHG.L–Cere4_5.L	-3.143	0.003	INS.L–Cere9.R	3.896	<0.001
LING.L–Verm3	-3.044	0.004	LING.L–Verm3	3.211	0.002

Note: FDR corrected, $p<0.05$. Abbreviations: sFC, state functional connectivity; AMYG.L, left amygdala; AMYG.R, right amygdala; ACG.L, left anterior cingulate and paracingulate gyri; CAL.L, left calcarine fissure and surrounding cortex; CAL.R, right calcarine fissure and surrounding cortex; CUN.R, right cuneus; Cere9.R, right cerebelum_9_; Cere4_5.L, left cerebelum_4_5_; INS.L, left insula; LING.L, left lingual gyrus; LING.R, right lingual gyrus; SFGdor.L, left superior frontal gyrus, dorsolateral; SFGmed.L, left superior frontal gyrus, medial.

Supplementary Table 11. One-way repeated measures ANOVA of Alterations in Variance of dFC in the control group (*n*=49)

Brain regions	Z1	Z2	Z3	*F*	*p*	ηp^2
SFGdor.L–ACG.L	0.016	0.022	0.033	7.772	0.001	0.097
REC.L–ACG.R	0.022	0.034	0.038	7.787	0.001	0.098
OLF.R–MTG.L	0.028	0.036	0.047	7.492	0.001	0.094
PHG.R–CRBL3.L	0.024	0.039	0.025	9.589	<0.001	0.118
PCG.R–CRBL6.L	0.033	0.053	0.035	7.551	0.001	0.095

Note: uncorrected, $p<0.001$. Z1, Z2 and Z3 indicated the mean values of variance of dFC before and after high-altitude exposure (Z1: Time 1; Z2: Time 2; Z3: Time 3). Abbreviations: dFC, dynamic functional connectivity; ACG.L, left anterior cingulate and paracingulate gyri; ACG.R, right anterior cingulate and paracingulate gyri; CRBL3.L, left cerebelum_3; CRBL6.L, left cerebelum_6; MTG.L, left middle temporal gyrus; OLF.R, right olfactory cortex; PHG.R, right parahippocampal gyrus; PCG.R, right posterior cingulate gyrus; REC.L, right gyrus rectus; SFGdor.L, left superior frontal gyrus, dorsolateral.

Supplementary Table 12. One-way Repeated Measures ANOVA of Alterations in dFC Variance with Paired-*t* test Results in the control group (*n*=49)

Time 1 *vs*. Time 2			Time 1 *vs*. Time 3		
Brain regions	*t*	*p*	Brain regions	*t*	*p*
PHG.R–CRBL3.L	3.877	<0.001	SFGdor.L–ACG.L	3.899	<0.001
-	-	-	REC.L–ACG.R	4.723	<0.001

Note: FDR corrected, $p<0.05$. Abbreviations: dFC, dynamic functional connectivity; ACG.L, left anterior cingulate and paracingulate gyri; ACG.R, right anterior cingulate and paracingulate gyri; CRBL3.L, left cerebelum_3; PHG.R, right parahippocampal gyrus; REC.L, right gyrus rectus; SFGdor.L, left superior frontal gyrus, dorsolateral.

Supplementary Table 13. Regions showing significantly altered GMV on ANOVA before and after High-altitude Exposure(n=49)

Brain region	MNI coordinates (mm)			Volume (mm^3)	Peak F value
	x	y	z		
PAL.R	24	-4.5	1.5	1265.621	11.698

Note: MNI coordinates, the coordinates of the peak point located in the MIN space. GRF corrected, voxel p<0.001, cluster p<0.01. Abbreviations: ANOVA, analysis of variance; GMV, gray matter volume; MNI, Montreal Neurological Institute; PAL, pallidum; R, right.

Supplementary Table 14. Regions showing significantly lower GMV on paired *t*-test before and after High-altitude Exposure(n=49)

Time 1 *vs*. Time 2						Time 1 *vs*. Time 3					
Brain region	MNI coordinates (mm)			Volume (mm^3)	Peak *T* value	Brain region	MNI coordinates (mm)			Volume (mm^3)	Peak *T* value
	x	*y*	*z*				*x*	*y*	*z*		
PAL.R	21	1.5	1.5	1032.753	-9.762	PAL.R	22.5	1.5	1.5	1265.621	-9.252

Note: MNI coordinates, the coordinates of the peak point located in the MIN space. GRF corrected, voxel p<0.001, cluster p<0.01. Abbreviations: MNI, Montreal Neurological Institute; GMV, gray matter volume; PAL.R, right pallidum.

Supplementary Figure 1. GMV of the PAL.R decreased during the 4-year HA exposure period. Compared with that pre-exposure, the blue areas indicate lower GMV in the participants after exposure. The color bar on the right indicates the *F* and *T* values. Abbreviations: GMV, gray matter volume; HA, high altitude; PAL, pallidum; R, right.

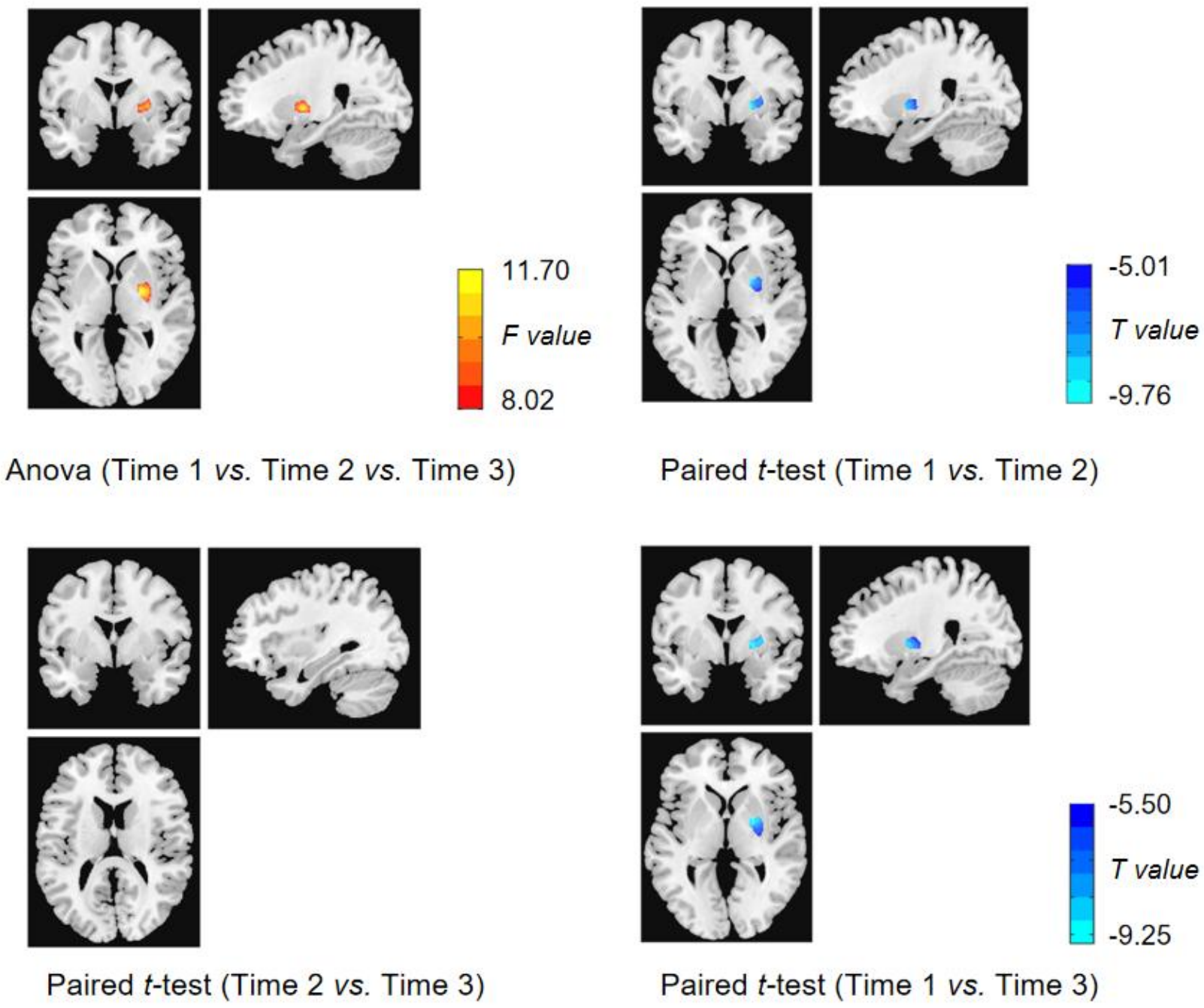

Supplement: Supplementary file 1 [file mmc1.pdf]
